# Supplementary material for: Risk–reward trade-off during carbon starvation generates dichotomy in motility endurance among marine bacteria
Source: Nat Microbiol. 2025 May 26;10(6):1393–403. doi: 10.1038/s41564-025-01997-7 (PMC12137127; doi:10.1038/s41564-025-01997-7)
Supplement: Supplementary file 1 — Supplementary Notes, Discussion, Tables 1–3 and 5, Table 4 caption and Figs. 1–3. [file 41564_2025_1997_MOESM1_ESM.pdf]

nature microbiology

Supplementary information https://doi.org/10.1038/s41564-025-01997-7

Risk–reward trade-off during carbon starvation generates dichotomy in motility endurance among marine bacteria

In the format provided by the authors and unedited

Contents

A Supplementary Notes **2**

1 Excluding the effect of alternative energy sources for motility . . . 2

1.1 Necromass recycling . . . 2

1.2 Light . . . 2

1.3 Trace levels of nutrients in starvation medium . . . 2

2 The potential role for storage compounds in biomass conversion . . . 3

3 Function of the selected features for genomic classification . . . 3

3.1 Resource conservation and fatty acid metabolism . . . 3

3.2 Oxidative stress defense . . . 3

3.3 Cysteine and methionine synthesis . . . 4

3.4 Motility and chemotaxis . . . 4

3.5 Capsule and biofilm formation . . . 5

4 Phylogenomic signature in the classifier . . . 5

B Supplementary Discussion **6**

1 The relevance for understanding bacterial search processes for the marine carbon cycle . . 6

2 Limokinetic motility as a strategy for oligotrophic waters . . . 6

3 How cells can optimize their exploratory behavior . . . 7

4 Chemotaxis . . . 7

5 Heterogeneity within limokinetic populations . . . 8

C Supplementary Figures **9**

D Supplementary Tables **13**

A Supplementary Notes

1 Excluding the effect of alternative energy sources for motility

1.1 Necromass recycling

We considered the recycling of necromass, the biomass of dead cells, as a potential mechanism providing energy for motility. Necromass recycling has been shown to be a possible mechanism to increase the survival of bacterial populations under prolonged starvation by attenuating the death rate of cells [49, 50]. However, in our starvation experiments the number of cells does not decrease over time for any of the strains, indicating that the net cell death, if any, is small (Fig. 3C). We cannot rule out that there is some initial cell death that is masked by the increase in the number of cells due to reductive divisions. Therefore, we measured cell death to estimate how much necromass is released during starvation.

Direct measurements of cell death using live/dead staining in the limokinetic and limostatic strains revealed a small difference in the fraction of dead cells between the two classes (Extended Data Figure 6). The fraction of dead cells decreased over time, which shows that necromass does not accumulate over the course of the starvation time and indicates that cell lysis occurs primarily at the onset of starvation. At the onset of starvation the average fraction of dead cells was 0.12 ± 0.04 and 0.23 ± 0.03 (mean $\pm$ one standard error of the mean), respectively, for limostatic and limokinetic strains. The difference between limostatic and limokinetic strains suggests limokinetic strains release 11 % more biomass in the form of necromass, which could in principle be available for swimming and fuel motility for approximately one day. However, this amount of biomass is much lower than decrease in biomass observed in limokinetic strains of 62 % over one week, showing that necromass does not act as long-term energy source for motility. Furthermore, the efficiency of biomass recycling ($\sim$20%, estimated in starvation experiments using *E. coli* [50]), indicates that the actual amount of necromass that can be re-absorbed into the population is even lower. We conclude that in our experiments biomass recycling plays at most a minor role in supplying cells with energy for motility.

1.2 Light

A potential alternative energy source during carbon starvation is light, which some bacteria can capture using proteorhodopsins. This strategy is widely used by oligotrophic non-motile bacteria, like SAR11 [93, 94]. Proteorhodopsins have also been proposed to provide energy for motility [95], a process that has been demonstrated in engineered (proton-motorized) *E. coli* [96]. Light-driven sodium pumps [97], a special class of rhodopsins, could potentially enable (sodium-motorized) marine bacteria to fuel motility using light. However, none of the 26 strains tested possesses proteorhodopsin genes. In addition, motility levels of *V. anguillarum* FS144 and *V. coralliilyticus* YB1 in the dark did not differ from those in the light (Extended Data Figure 6g,h). Together, these data indicate that the energy source for limokinetic cells during starvation does not come from light.

1.3 Trace levels of nutrients in starvation medium

Additional experiments confirmed that the energy source that powers motility under starvation is internal to the cells. The chemical energy for swimming could be external to the cells (e.g., provided by low levels of residual nutrients in the medium) or internal (e.g., resulting from the conversion of biomass to energy). These two sources would be expected to yield different relationships between the motility level and the concentration of cells. An external energy source implies decreasing energy for motility per cell with increasing cell concentration, as cells must share the available nutrients, whereas for an internal energy source the energy for motility per cell would be independent of the cell concentration. To distinguish between these two possibilities, we measured the dependence of motility on cell concentration for four limokinetic strains under starvation. We prepared a dilution series of cell concentration spanning two orders of magnitude and quantified the fraction of motile cells for at least 24 h following the onset of starvation. A linear fit to the motile fraction as a function of the logarithm of the relative cell concentration reveals a positive slope for all strains, with average slope of 0.08 ± 0.04 (Extended Data Figure 6i), thereby excluding a negative dependence of the motile fraction on cell concentration. Together with the decrease in biomass (Fig 3), this result shows that the energy source to power swimming during starvation in limokinetic cells is internal to the cells.

2 The potential role for storage compounds in biomass conversion

To understand the nature of the biomass that is converted into energy, we investigated whether limokinetic strains accumulate energy storage compounds before starvation. Using four limokinetic and three limostatic strains in carbon-replete media, we assayed the presence of polyphosphate (polyP) using DAPI staining and polyhydrobutyrate (PHB) using Bodipy staining. Both storage compounds have been suggested to act as energy sources for motility [98, 99]. We observed no formation of polyP granules in carbon replete media, except in a minority of cells in limokinetic strain *Alteromonas* sp. 4B03 (Extended Data Figure 7a-c). In contrast, two out of three limokinetic strains produced considerable amounts of PHB, whereas limostatic strains produced either no or lower amounts of PHB (Extended Data Figure 7d,e). This suggests a potential role of storage compound PHB as an additional yet not exclusive energy source of motility in limokinetic strains during starvation. Another bacterial storage compound is glycogen, for which many limokinetic and limostatic strains have synthesis genes (Supplementary Table S2) but it is unclear if glycogen can act as an energy source over extended times [100, 101]. Finally, bacteria may catabolize biomass that does not have a specific storage function, such as membrane lipids or ribosomes, for energy [7].

3 Function of the selected features for genomic classification

3.1 Resource conservation and fatty acid metabolism

A small number of genes associated with limokinetic strains are involved in energy or resource conservation. Limokinetic strains convert biomass to energy to fuel motility (Main text, Fig. 3). A possible energy source for cells are lipids, a high-energy component of cellular biomass. A number of accessory genes involved with fatty acid degradation were selected for the classifier. The presence of malonate semialdehyde dehydrogenase (K00140; *mmsA*) and inorganic pyrophosphatase (K01507; *ppa*) seems to indicate an important role for efficient fatty acid degradation in these cells, as both are downstream metabolites formed from the undesirable leftovers of beta-oxidation (propanoyl-CoA and pyrophosphate) and these enzymes help to confer complete degradation of these byproducts. The presence of a short-chain fatty acid transporter (K02106,*atoE*) and glyoxylate/hydroxypyruvate reductase (K12972; *ghrA*) could also allow for the uptake and use of small carboxylic acids from the water column. The selected set also includes electron transfer flavoproteins (K03521; *etfA*), which efficiently couple the production of $FADH_2$ from beta-oxidation to the ubiquinone pool of the electron transport chain, linking fatty acid degradation directly to the production of ATP [102].

3.2 Oxidative stress defense

Many features of our classifier relate to reactive oxygen species (ROS) and related oxidative stress. ROS [103] are abundant in marine environments [104, 105] and lead to oxidative stress, which is believed to be especially detrimental for cells in situations that permit limited or no growth [106, 107], because the ability to regenerate damaged proteins or cellular machinery is limited. Limokinetic strains are likely experiencing greater oxidative stress during starvation than limostatic strains, as motility requires an increased metabolism and respiration level, which in turn generates ROS and increases ROS-associated cell damage [108].

The strongest direct evidence for a strategy of mitigating oxidative stress is the presence of catalase-peroxidase *katG* (K03782) as one of the most highly weighted features our set associated with limokinetic strains. Another protein in our feature set, Dps (K04047), is a DNA-binding protein that is typically expressed under starvation conditions and non-specifically binds DNA, forming a ROS-resistant structure [109, 110, 111]. Other indirect or systemic adaptations are also present, such as phosphogluconate dehydratase (K01690; *edd*), indicating a preference for the Entner–Doudoroff pathway (EDP), for which it acts as the entry point. The EDP has been shown to be preferentially used over the Embden–Meyerhof–Parnas pathway (EMP) in a number of marine bacteria as it appears to confer additional resistance to oxidative stress [112]. K09797 is also present and represents a protein of unknown function, but homologous proteins have been annotated as ‘oxidative stress response proteins’ in a number of other bacteria.

To test if the two groups of strains showed different sensitivities to ROS stress, we measured the effect of ROS on selected strains from each group by exposing them to hydrogen peroxide. When we applied hydrogen peroxide during exponential growth, we measured no significant difference between limokinetic and limostatic strains in the growth rate or motility level (Extended Data Figure 8). We

then applied different levels of hydrogen peroxide oxidative stress to cells starved for 24 h and measured the lag time of growth resumption after nutrients were once again provided (Extended Data Figure 8). These experiments revealed a mildly elevated increase in sensitivity of limostatic cells to oxidative stress compared to the lag time without added hydrogen peroxide (on average 1.36-fold vs. 1.09-fold), but the oxidative stress level ($[H_2O_2] = 10$ μM) at which this effect occurred was higher than cells would experience in nature. Typically, oxidative stress in marine environments is expected to be low in intensity ($[H_2O_2] \approx 100$ nM) but long-lasting [113]. Therefore, it is possible that differences between the two classes are only apparent when the effects of oxidative stress accumulate on longer timescales than investigated in this study, for example through DNA and protein damage.

Finally, it is possible that the presence of ROS-related genes are a reflection of the community context of the bacterial cells. It has been shown that cyanobacteria can 'offload' the cost of ROS detoxification to algal cells that have to perform this function anyway [114], an example of the black queen hypothesis [115]. As limostatic strains rapidly lose motility, they likely prevail in situations where the distance between particles is small, such as algal blooms, where the community provides protection against ROS. By contrast, limokinetic strains, must provide their own ROS detoxification machinery in order to survive in scenarios with low particle (and algal) concentrations.

3.3 Cysteine and methionine synthesis

Another series of genes associated with the limokinetic response are involved in synthesis of sulfur-containing amino acids, such as methionine and cysteine. MetX (K00641) catalyzes the synthesis of the homocysteine and methionine precursor acetyl-L-homoserine. This is less common alternative to the more conventional route via succinyl-L-homoserine commonly found in Gammaproteobacteria [116]. Reasons for preferring one pathway over the other are not known, but it could be speculated that the use of the acetyl-L-homoserine pathway constitutes a slight fitness advantage during starvation, as it requires fewer enzymes and is able to incorporate sulfide directly without the use of a cysteine intermediate (cystathionine). Also associated with homocysteine and methionine metabolism, the *cysG* (K02302) gene encodes a multifunctional enzyme that acts as a methyltransferase implicated in both sirohaem and vitamin B12 synthesis [117]. Importantly, sirohaem is a required cofactor for assimilatory sulfite reductases that produce hydrogen sulfide to be used in homocysteine and methionine synthesis [118]. The *cysG* gene also functions in cobalamin (B12) maturation and its presence may represent the importance of B12 synthesis in this regard [117] - methylation of homocysteine is the final step in methionine biosynthesis and B12-utilizing enzymes (metH) for this reaction have been shown to have a ∼30-fold increase in activity over B12-independent proteins[119]. Overall, the presence of *cysG* and related genes is likely due to an overall heightened requirement for sulfur-containing amino acids. One possibility for an increased demand of these amino acids is a demand for methionine to act as a methyl-donating molecule in chemotactic cells [80]. Another possibility is that this is also related to protein repair in response to oxidative stress, as cysteine and methionine are reported to be most sensitive to ROS damage [120]. Cysteine is also a precursor for the production of glutathione, the main ROS-scavenging molecule in the cell, suggesting that the genes involved in cysteine and methionine synthesis could play additional roles in oxidative stress defense.

3.4 Motility and chemotaxis

A number of motility-related regulatory genes are implicated as features of the limokinetic strains. Included among these are two-component regulatory systems for the expression of flagella under quorum sensing [121] (K07666, K07645; *qseB, qseC*) or swarming [122] (K20977, K20978; *hsbR, hsbA*) scenarios. It is likely that these are in some way responsible for triggering the decision to swim in these cells and they could be potential targets for future studies into the mechanism underlying the limokinetic–limostatic distinction. Accessory proteins such as CheC and CheD (K03410,K03411) are also present. In *B. subtilis*, CheC and CheD have been shown to form an alternative mechanism of sensory adaptation that works alongside methylation-dependent adaptation (albeit with a likely lower dynamic range) [123]. Sensory adaptation through methylation is an energetically expensive trait, equivalent to approximately 10% of the cost of turning the flagellar motor [124]. This means that cells might favor using CheCD for sensory adaptation in situations of starvation, where energy or methyl-group donor molecules (like methionine, see 'Cysteine and methionine synthesis' above) are scarce [80].

3.5 Capsule and biofilm formation

Capsular polysaccharides can protect against adverse environmental conditions including desiccation or osmotic stress, unfavorable pH, the presence of antibacterial compounds, viral predation, and oxidative stress, and are common in marine bacteria [125]. The production of capsular polysaccharides has been shown to confer fitness advantages in gammaproteobacteria under nutrient-limited conditions [126]. The *vpsO, vpsM* and *vpsN* genes involved in capsular polysaccharide synthesis and export are present in our final set of classifier features (K16554, K20920, K20988), as is *wcaJ* (undecaprenyl-phosphate glucose phospotransferase; K03606), which is required for the first step in capsule formation, linking sugar residues to a lipid anchoring moiety [127, 128]. Also related to capsule formation, CDP-4-dehydro-6-deoxyglucose reductase (K00523) produces precursors for O-antigen/Lipopolysaccharide production, which has been shown to confer resistance to a variety of environmental stressors [129, 130].

Several capsule polysaccharide genes are also involved in extracellular polysaccharide production of Vibrio (exo)polysaccharide (VPS) biofilms [131]. Within the Vibrionaceae, the possession of VPS genes can be used as a predictor of limokinetic behavior (Supplementary Table 5). Interestingly, we discovered that the VPS-assocated gene rbmC (not part of the classifier) can correctly predict limokinetic behavior within the Vibrionaceae (accuracy 93%, when including all the Vibrio species in this study; Supplementary Table 5). RbmC has been shown to facilitate linking of VPS-biofilm to host-produced glycans such as mucus [132], and could thereby assist host colonization by marine bacteria. This suggests limokinetic behavior could play a role in pathogenicity and symbiosis [133, 134].

4 Phylogenomic signature in the classifier

Because there is a taxonomic signature in our data (26% of the Vibrionaceae family are limokinetic, compared to 86% of strains from other families; Extended Data Figure 1), we considered the effect of phylogeny on feature selection using *post-hoc* phylogenetic logistic and linear regression analyses of classification outcome against OG presence. We focused first on if the feature (Orthologous group) could be explained by phylogeny. This was done to examine if the patterns we observed are due to only closely related bacteria having similar traits, rather than a broader association across the more divergent bacteria in our dataset. First, the binary outcome of both the limokinetic as well as the limostatic classifier were regressed against each individual KEGG ortholog presence/absence in all genomes using a generalized linear model that did not consider phylogeny in the logistic regression. We then included a whole genome phylogeny in a separate phylogenetic generalized linear model framework to assess if phylogeny could explain any observed association as implemented in the R package phylolm [90] by comparing the statistical significance of the slope coefficient in the two regression models.

One well-known disadvantage of logistic regression occurs when near-complete separation of genomic features and the classification outcome occur, leading to a large uncertainty of the coefficient estimates [135] (Fig. 4a). Therefore, features where the logistic regression without including phylogeny were insignificant were further examined with alternative phylogentic comparative methods: Pagel's method for correlated binary trait evolution [136] as implemented in the phytools R package [137] and phylogenetic linear regression on the binary variables using Pagel's lambda as implemented in the R package phylolm [90]. This confirmed most features to be a robust feature of the dataset ($p < 0.10$ for at least 2 test), but the limokinetic classifier contains six features where an influence of phylogeny cannot be excluded (K02106,K12972,K00641,K00523,K03410,K03411) (Supplementary Table 3).

Finally, we performed a complimentary analysis by combining all of the genomic features using phylogenetic principal components analysis [137] and assessed if the orthogonal combinations of features in the classifier (principal component scores for each genome) were associated with the Bayesian classifier outcome using the same phylogenetic logistic regression approach outlined above. For the limokinetic classifier, the first 6 principal component axes of phylogenetic PCA explain 89.2 % of the total variation in the dataset. PC1 explains 39.3 % of total variation is highly significant when regressed against classification outcome and when including phylogeny in the model ($p = 0.008$). The 5 strongest loadings on this principal component in order are K07666, K07645, K01507, K03411, K20977. For the limostatic classifier, the first 6 principal component axes of phylogenetic PCA explain 70.5 % of the total variation in the dataset. PC1 explains 26.1 % of total variation and is highly significant when regressed against classification outcome and including phylogeny in the model ($p = 0.009$) and the 5 strongest loadings (an arbitrary cutoff) on this principal component in order are: K12113, K04015,K12265,K12264,K09758. These analyses further confirm that when these features are considered together their relationship to classification outcome cannot be explained by phylogeny alone.

We then investigated the consequences of phylogenetic bias in classifier prediction. The collection used to train the classifier consists of 19 strains from the Vibrionaceae family (the genera *Vibrio* and *Aliivibrio*), of which the majority (14) are limostatic, and 7 strains from other families (3 Alteromonadaceae, 2 Oceanospirillaceae and 2 Phyllobacteriaceae), of which the majority (6) are limokinetic (Extended Data Figure 1, Supplementary Table S1). Therefore, a taxonomic model that classifies all Vibrionaceae strains as limostatic and all others as limokinetic would accurately predict the behavior of 20/26 strains (77%), significantly different from a binomial random classifier ($p = 0.004$, calculated as the probability of correctly predicting at least 20 of 26 strains, assuming a binomial distribution with 50% as the chance of a successful prediction for each strain). If we include the 7 additional strains used for prediction, this decreases slightly to 25/33 correct (75%). The Bayesian limokinetic and limostatic classifiers built in this work (Fig. 4) both correctly predict 23/26 and 28/33 of the strains (88%), outperforming the phylogenetic classifier ($p = 0.07$, the chance of predicting at least 28 out of 33 strains correctly, with a success chance for each strain of 75%). Within all Vibrionaceae in this study, the limokinetic classifier correctly predicts 20/23 strains. This is better than a phylogenetic approach ($p = 0.05$, based on the chance of predicting at least 20/23 correctly with an individual chance of 16/23 = 69.5%). Hence, in this dataset there appears to be a taxonomic component associated with the limokinetic/limostatic dichotomy, but this classifies the strains less accurately than a Bayesian classifier built upon genomic information.

B Supplementary Discussion

1 The relevance for understanding bacterial search processes for the marine carbon cycle

The association of marine bacteria with nutrient hotspots affects important oceanic elemental fluxes. An important type of hotspots are sinking marine particles, consisting of organic matter fixed by primary producers, that sink to the bottom of the ocean and thereby effectively sequester carbon from the atmosphere [5]. However, copiotrophic marine bacteria colonize and degrade these particles before they reach the ocean floor, and are responsible for up to 29 % of the carbon flux attenuation [138] and act as gatekeepers of the vertical carbon flux [52, 53]. In many areas, such as in the open ocean [26], particles are very dilute and search times can extend to days or weeks. There has been considerable effort in obtaining a more mechanistic understanding of the exploitation of the organic matter in marine particles [139, 24, 140], but the degradation process ultimately hinges on the successful colonization of these particles, where low number-fluctuations in inoculi can create strongly divergent bacterial communities on particles [141]. Unsuccessful colonization is suggested to be a key driver for some particles escaping degradation [54]. Therefore, a better understanding of the bacterial search process for particles may better constrain models of the microbial contribution to carbon flux in the ocean.

2 Limokinetic motility as a strategy for oligotrophic waters

75 % of the open ocean are oligotrophic [142], having background concentrations of dissolved organic matter (DOM) insufficient to sustain the growth of most copiotrophic bacteria [143, 144, 6]. A comparison of carbon concentrations and uptake rates show that DOM concentrations in oligotrophic waters are also typically insufficient to sustain swimming. For example, a concentration of serine of 100 nM would generate an energy flux of $\sim 10^5$ ATP/s (based on a measured bacterial uptake rate of $1 \cdot 10^5$ molecules/cell/s [145, 146], a K_M of 1 µM [147], and an energy equivalent per serine molecule of 12 ATP [148]). Thus, background concentrations of amino acids of 100 nM [149] measured in coastal regions of the ocean are expected to be sufficient to fuel swimming with a velocity of 60 µm/s (requiring $\sim 9 \cdot 10^4$ ATP/s, as calculated using the equation and parameters presented in the main text), but this would severely reduce the energy flux available for cellular maintenance. Lower concentrations of nutrients are likely insufficient to fuel swimming, although some copiotrophs can express highly specific transporters under starvation with values of K_M as low as 10 nM [150]. However, this is unlikely to generate a large energetic flux as models of high-affinity transporters show that there is a trade-off between affinity and the maximum uptake rate [151]. Therefore, in some coastal regions the background nutrient concentrations in the ocean may provide sufficient energy to swim, but in most regions of the ocean bacteria exposed to the background concentrations of nutrients will lack sufficient nutrients to fuel swimming.

Typical search times of motile marine bacteria for organic matter particles are expected to be on the order of hours to weeks [26]. In the areas with highest primary productivity (Beaufort Sea), the

concentration of particles reaches $c = 50{,}000$ particles/L (data from Tara oceans [152], integrating over all particle sizes from 20 μm to 1 mm), which corresponds to a typical distance of $L = 1.5$ mm between particles ($L \approx 0.553c^{-1/3}$ [153]). Using the relation $t = L^2/6D$ for the time t required to diffuse a distance L, and assuming a diffusion coefficient of $D = 1 \cdot 10^{-6}$ cm^{2}s^{-1}, this corresponds to a search time t of approximately 1 hour. By contrast, a non-motile bacterium $D \approx 1 \cdot 10^{-8}$ would have a search time of 4 days [26]. In such particle-rich environments, a small fraction of limostatic strains may be able to find a new particle before motility ceases, whereas limokinetic strains are expected to easily find a new particle. The discrepancy between the two strategies increases as the particle concentration decreases. In areas with very low productivity the particle concentration is typically very low. For example in the South Pacific Ocean, at $c = 0.4$ particles/ L, corresponding to a typical distance of $L = 74$ mm between particles, the average search time t for motile cells is more than 3 months. Given our measurements of the cost of bacterial motility (62 % biomass reduction over one week), it seems unlikely that motility endurance extends for multiple months. However, assuming exponentially distributed search times [26], even in this extremely oligotrophic environment 10% of the motile bacteria will have encountered a particle within one week. This indicates that the limokinetic strategy may still be viable in extremely nutrient-poor regions of the ocean, even when the average search time significantly exceeds the average swimming endurance time of the population.

3 How cells can optimize their exploratory behavior

The encounter rate of bacteria with larger particles scales linearly with the effective diffusion coefficient that describes the bacteria's random walk [25]. Hence, the effective diffusion coefficient of bacteria is a quantitative measure of how much space they explore. The average diffusion coefficient D of the motile fraction of a population performing a random walk by run-reverse-flick can be estimated from measured bacterial trajectories, according to $D = (1/6)v^2(R + 4D_R)/(R + 2D_R)^2$ [154], where v is the average velocity of motile cells (Extended Data Figure 6a), R is the reorientation frequency (Extended Data Figure 6b), and $D_R = 0.035\text{rad}^2/\text{s}$ is the rotational diffusion coefficient [155]. Because the typical reversal frequencies (2-3 s^{-1} are much higher than the rotational diffusion D_R, the relation can be simplified to $(1/6)v^2/R$, which is also the effective diffusion coefficient for cells performing a run-reverse random walk (without flicks) [154], which is the prevailing random walk of single-flagellated bacteria at low velocities [32]. Hence we can use this expression to approximate the effective diffusion coefficients of the cell's random walk. The average effective diffusion coefficient of all limokinetic strains decreased from $1.5 \pm 0.5 \cdot 10^{-6}$ cm^{2}s^{-1} in nutrient-rich media to $0.8 \pm 0.4 \cdot 10^{-6}$ cm^{2}s^{-1} during starvation (t-test: $p = 0.003$).

Starved cells would benefit from increasing the amount of space they explore in search for new resources, i.e., their diffusion coefficient. They can do so by either increasing their swimming velocity v, which is energetically costly, or by lowering their reorientation rate (i.e., decreasing R), which comes at no energetic cost and would thus be a preferable adaptation. However, tracking showed that, rather than decreasing, the average reorientation frequency increases during starvation (Extended Data Figure 6b). This suggests that there might be additional reasons for cells not to decrease their reorientation frequency, for example chemotaxis (Supplementary Discussion 5).

The possibility that bacteria under starvation adopt forms of random walk that enable more exploration of space, and thus more encounters, cannot be ruled out from our experiments, but would require longer trajectories that are challenging to obtain. One such possibility would be Lévy-flights [156, 157] with infrequent runs that much longer than the average run length. Another way for bacterial cells to enhance diffusion without additional cost would be an asymmetry in swimming velocities between forward and reverse runs, as has been shown to boost diffusion coefficients of lophotrichous bacteria by several fold [158, 159].

4 Chemotaxis

This study has focused on the effect of starvation on motility alone, but what is the role of chemotaxis in the dichotomy? In the typical resource landscape of the ocean, hotspots are characterized by local gradients that marine bacteria must first find, before they can use chemotaxis to climb the gradient and home in on the hotspot: it is swimming per se (i.e., 'random motility'), rather than chemotaxis, that provides the largest boost (100- to 1000-fold; [26]) in the encounter rate with such hotspots over non-motile bacteria. To increase encounters further, bacteria could either increase their swimming velocity, which is energetically costly, or suppress reorientation events (since this results in exploration of a larger

volume [154], see Supplementary Discussion 3), which comes at no energetic cost. Our data reveal no such suppression: the average reorientation frequency of all limokinetic strains increased from 2.3±0.4 s^{-1} in nutrient-rich media to 3.3±0.8 s^{-1} after two days of starvation (t-test: $p = 0.002$, Extended Data Figure 6, Supplementary Table 1). Because reorientations are crucial for chemotaxis, a decrease in reorientation frequency would come at the cost of reduced responsiveness to short-lived chemical gradients [154, 160], which are typical in the marine environment [10, 161]. All 26 tested strains possess chemotaxis genes and both classes include species that are highly chemotactic, including for example *Vibrio anguillarum* [161] and *Vibrio alginolyticus* [155]. The fact that limokinetic strains do not suppress reorientations during starvation, despite the benefit this would provide in searching for hotspots, suggests that maintaining the ability to perform chemotaxis also during starvation is important. Furthermore, several limokinetic strains possess an alternative adaptation system (CheC, K03410 and CheD, K03411; Fig. 4A) that requires less resources and therefore could be beneficial for chemotaxis during starvation (Supplementary Text).

5 Heterogeneity within limokinetic populations

Even in limokinetic populations, the average motile fraction per strain during starvation varied from 0.05 to 0.55, and hence a considerable fraction of the cells were not swimming. One reason could be that limokinetic strains are hedging their bets, where the non-motile fraction of the population preserves resources while the motile fraction searches. Alternatively, it is possible that cells in limokinetic strains are only motile a fraction of the time. Many species of bacteria are able to pause motility without losing flagella [162, 18, 163], likely through a 'clutch-like' mechanism [164, 165]. Our observation that during starvation the flagellated fraction of cells in limokinetic strains is higher than the motile fraction indicates that these strains are also capable of pausing motility. However, the duration of these pauses, and the degree to which they are present in all of our strains, cannot be reliably determined in our data and requires tracking methods capable of measuring the motility of individual cells over prolonged times. Furthermore, such experiments may reveal further variation in the motility and dispersal strategies of limokinetic strains.

C Supplementary Figures

Figure S1: **Supplementary information on cell tracking a**: (left) Example nonmotile cell of strain F3R08 without background correction. (middle) Intensity profile along the red line in the example panel for both the raw data and a background-corrected signal. (right) The normalized maximum intensity of raw and background-corrected data points. The average value is 0.99, indicating that the background correction only marginally affects peak intensity. Data from Supplementary Video S11. **b**: Cellular velocity distributions for 22 strains during growth in carbon-replete media, with a bin width of 3 μm/s. Each distribution originates from a single tracking experiment. A threshold of 12 μm/s (dashed lines) was used to distinguish motile from non-motile cells in the population. **c**: Effect of residence time in observation chambers on motility parameters: motile fraction (left panel), average velocity of motile cells (middle panel) and reorientation frequency (right panel). All data are from a single biological replicate per strain.

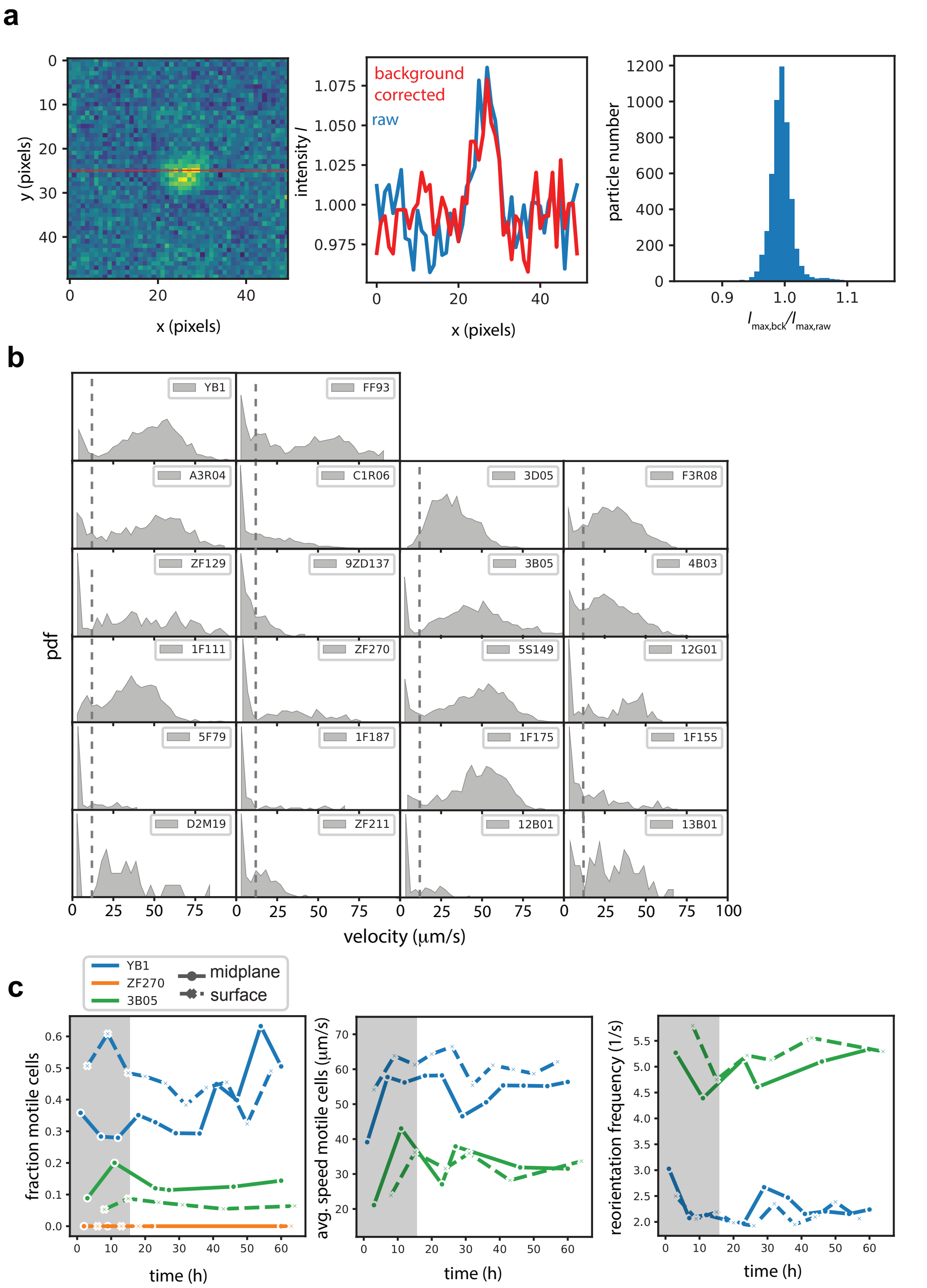

Figure S2: Gating strategy used for flow cytometry measurements. A: Bacterial cell count were determined by applying gating values on the Forward Scatter (FSC-A) and SYBR Green fluorescence (FITC-A). B: Cell counts before (red) and after (blue) applying gating criteria.

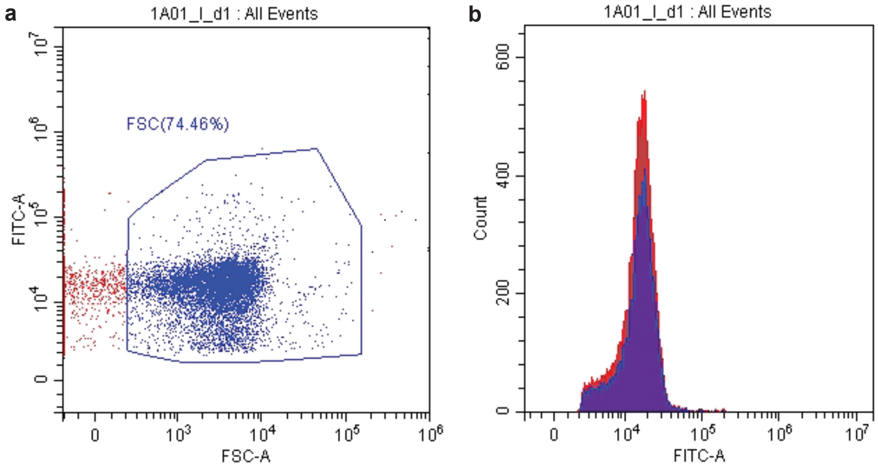

Figure S3: Recursive feature elimination (RFE) for the training set of our limokinetic Bayesian classifier (green) compared to the mean (red) and mean plus standard deviation (orange, dashed) of 100 trials in which class (limostatic/limokinetic) was randomly assigned. RFE was run for combinations of 2, 4, 8, 16, 32, 64, 128, 256, 512, and 1000 features.

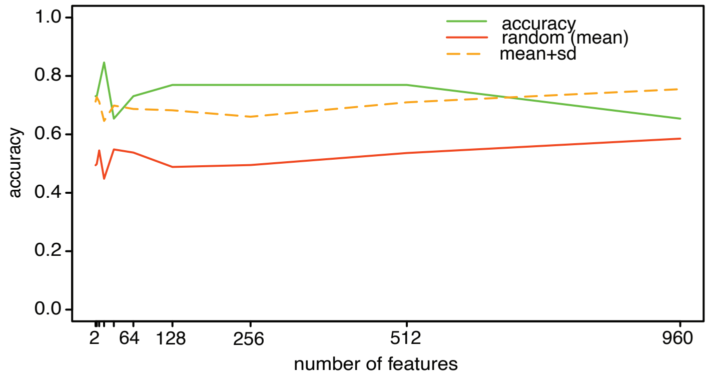

D Supplementary Tables

Supplementary Table 1: Motility parameters for each strain during growth in carbon-replete medium (g) and during carbon starvation* (s). Fraction of motile cells f , reorientation frequency R (1/s), and average swimming velocity of motile cells $\langle v \rangle$ (µm/s). Since velocities are computed from trajectories in 2D, absolute velocities can be underestimated by up to 30 % [166].

Strain	Species	accession	source	Category	f_g	f_s	$\langle v \rangle_g$	$\langle v \rangle_s$	R_g	R_s
ZF211	*Aliivibrio fischeri*	GCF_000287175.2	[70]	Limostatic	0.07	0.000	19	–	5.5	–
D2M19	*Marinobacter salexigens*	GCF_018860765.1	[139]	Limostatic	0.18	0.000	27	–	4.1	–
13B01	*Vibrio splendidus*	GCF_001691275.1	[70]	Limostatic	0.59	0.005	31	–	1.8	–
12B01	*Vibrio splendidus*	GCF_000152765.1	[70]	Limostatic	0.40	0.012	37	–	2.3	–
1A01	*Vibrio splendidus*	GCF_002700025.1	[29]	Limostatic	0.51	0.001	43	–	1.6	–
FF-500	*Vibrio splendidus*	GCF_000272265.2	[70]	Limostatic	0.49	0.007	39	–	1.1	–
5F79	*Vibrio lentus*	GCF_001691195.1	[70]	Limostatic	0.14	0.015	25	–	2.4	–
1F187	*Vibrio tasmaniensis*	GCF_000272405.2	[70]	Limostatic	0.10	0.012	27	–	3.7	–
1F155	*Vibrio tasmaniensis*	GCF_000272385.2	[70]	Limostatic	0.41	0.020	33	–	2.2	–
ZF270	*Vibrio cyclitrophicus*	GCF_000256465.2	[70]	Limostatic	0.55	0.014	43	–	2.5	–
1F111	*Vibrio cyclitrophicus*	GCF_000247005.2	[70]	Limostatic	0.56	0.01	33	–	1.5	–
1F175	*Vibrio cyclitrophicus*	GCF_000256135.2	[70]	Limostatic	0.61	0.01	46	–	1.6	–
12G01	*Vibrio alginolyticus*	GCF_000153505.1	[70]	Limostatic	0.24	0.01	30	–	2.5	–
5S149	*Vibrio kanaloae*	GCF_000272165.2	[70]	Limostatic	0.61	0.02	46	–	2.0	–
9ZD137	*Vibrio sp. F10*	GCF_000287015.2	[70]	Limostatic	0.40	0.02	30	–	2.5	–
4B03	*Alteromonas sp.*	GCF_018619335.1	[29]	Limokinetic	0.60	0.048	38	22	2.6	3.5
ZF129	*Vibrio sp.*	GCF_000287055.2	[70]	Limokinetic	0.42	0.062	50	26	1.9	3.6
3B05	*Neptunomonas phycophila*	GCF_018619275.1	[29]	Limokinetic	0.72	0.080	51	36	3.4	3.6
C1RO6	*Amphritea atlantica*	GCF_018860855.1	[139]	Limokinetic	0.63	0.110	33	21	2.4	3.9
A3R04	*Aestuariibacter sp.*	GCF_018860805.1	[139]	Limokinetic	0.57	0.150	52	34	2.0	3.2
F3R08	*Marinobacter sp.*	GCF_018860425.1	[139]	Limokinetic	0.67	0.25	36	27	2.2	2.9
3D05	*Pseudoalteromonas sp.*	GCF_002723455.1	[29]	Limokinetic	0.57	0.33	31	35	2.3	2.2
FS144	*Vibrio anguillarum*	GCF_000287115.2	[70]	Limokinetic	0.67	0.25	50	38	2.1	2.4
12B09	*Vibrio anguillarum*	GCF_000287135.2	[70]	Limokinetic	0.52	0.290	45	37	2.2	2.8
FF93	*Vibrio anguillarum*	GCF_000287095.2	[70]	Limokinetic	0.49	0.370	43	33	1.9	2.4
YB1	*Vibrio coralliilyticus*	GCF_000176135.1	[?]	Limokinetic	0.80	0.550	43	53	2.5	2.4
	average ± std **			All	0.48±0.19	0.10±0.15	37±9	–	2.44 ±0.87	–
	average ± std			Limostatic	0.39±0.20	0.01±0.01	33±8	–	2.54 ±1.08	–
	average ± std			Limokinetic	0.61±0.11	0.23±0.16	43±7	33±9	2.30 ±0.42	3.0±0.6

*:for all averages during starvation, all times >1 h are included.
**:average and standard deviation of the average number per strain.

Supplementary Table 2: Presence (+) or absence (+) of the main storage compound synthesis and catabolysis genes for each species, based on annotated genomes (accession numbers in Supplementary Table S1) and annotations according to BioCyc [167]. Presence/absence are the same for all strains of the same species unless noted otherwise.

Species	Category	PHB			PolyP		glycogen	
		phaB	*phaC*	*phaZ*	*ppk1*	*ppk2*	*glgC*	*glgP*
Aliivibrio fischeri	Limostatic	-	-	-	-	-	+	+
Marinobacter salexigens	Limostatic	+	+	-	+	-	-	-
Vibrio splendidus	Limostatic	+*	+*	-	+	-	+	+
Vibrio lentus	Limostatic	+	+	-	+	-	+	+
Vibrio tasmaniensis	Limostatic	-	+	-	+	-	+	+
Vibrio cyclitrophicus	Limostatic	+	+	-	+**	-	+	+
Vibrio alginolyticus	Limostatic	+	+	-	+	+	+	+
Vibrio kanaloae	Limostatic	+	+	-	+	-	+	+
Vibrio sp. F10	Limostatic	+	+	-	-	-	+	+
Alteromonas sp. 4B03	Limokinetic	-	-	-	+	+	+	+
Vibrio sp. ZF-129	Limokinetic	+	-	-	-	-	+	+
Neptunomonas phycophila	Limokinetic	+	+	+	+	+	-	-
Amphritea atlantica	Limokinetic	+	+	-	+	-	-	-
Aestuariibacter sp.	Limokinetic	+	+	+	+	+	+	+
Marinobacter sp.	Limokinetic	+	+	+	+	+	+	+
Pseudoalteromonas sp.	Limokinetic	-	-	-	+	+	+	+
Vibrio anguillarum	Limokinetic	-	-	-	+	-	+	+
Vibrio coralliilyticus	Limokinetic	+	+	-	+	+	+	+

* *phaC* absent in 12B01 only, *phaB* absent in strains FF-500 and 13B01.
** *ppk1* absent in strain 1F111

Supplementary Table 3: Results of regression analysis with and without phylogeny for limokinetic classifer. For each feature, different models were applied: logistic regression (log), logistic regression with phylogeny (plog), a linear model with phylogeny (plin) and Pagel's binary character correlation test (Pagel). For each model the slope and intercept (-icpt) are reported with the corresponding p-value in brackets, except Pagel where only a p-value is reported.

Feature	Log-slope	Log-icpt	plog-icpt	plog-slope	plin-icpt	plin-slope	Pagel
K00140	20.66 (0.99)	-1.09 (0.05)	-0.01 (0.98)	2.76 (0.07)	0.76 (0.00)	0.18 (0.14)	0.03
K00523	20.25 (0.99)	-0.69 (0.16)	0.35 (0.71)	2.10 (0.18)	0.81 (0.00)	0.13 (0.30)	0.09
K00641	20.25 (0.99)	-0.69 (0.16)	0.32 (0.73)	2.20 (0.17)	0.56 (0.05)	0.41 (0.08)	0.08
K01507	23.05 (0.99)	-2.48 (0.01)	-1.02 (0.27)	4.60 (0.01)	0.22 (0.06)	0.84 (2.78e-10)	0.00
K01690	20.95 (0.99)	-1.38 (0.03)	-0.26 (0.76)	3.26 (0.04)	0.47 (0.06)	0.50 (0.00)	0.03
K02106	18.97 (0.99)	-0.40 (0.37)	0.57 (0.55)	1.61 (0.32)	0.81 (0.00)	0.16 (0.53)	0.15
K02302	20.95 (0.99)	-1.38 (0.03)	-0.12 (0.88)	3.29 (0.05)	0.52 (0.01)	0.54 (0.00)	0.03
K03410	18.97 (0.99)	-0.40 (0.37)	0.81 (0.33)	1.75 (0.31)	0.87 (0.00)	0.42 (0.02)	0.33
K03411	20.44 (0.99)	-0.87 (0.10)	0.47 (0.58)	2.95 (0.14)	0.77 (2.35e-05)	0.65 (2.07e-05)	0.21
K03521	20.44 (0.99)	-0.87 (0.10)	0.13 (0.88)	2.58 (0.11)	0.48 (0.07)	0.50 (0.01)	0.04
K03606	4.18 (0.00)	-1.70 (0.02)	-0.14 (0.88)	2.88 (0.03)	nan (nan)	nan (nan)	nan
K03782	22.35 (0.99)	-1.79 (0.01)	-0.42 (0.61)	3.57 (0.02)	0.71 (0.00)	0.28 (0.00)	0.00
K04047	20.95 (0.99)	-1.38 (0.03)	-0.12 (0.88)	3.29 (0.05)	0.52 (0.01)	0.54 (0.00)	0.03
K07645	20.95 (0.99)	-1.38 (0.03)	0.09 (0.91)	3.68 (0.08)	0.66 (5.21e-05)	0.65 (1.09e-06)	0.06
K07666	51.13 (0.99)	-25.56 (0.99)	-3.21 (0.03)	6.58 (0.00)	nan (nan)	nan (nan)	0.00
K09797	20.66 (0.99)	-1.09 (0.05)	0.19 (0.82)	2.97 (0.09)	0.61 (0.00)	0.57 (0.00)	0.08
K12972	18.97 (0.99)	-0.40 (0.37)	0.57 (0.55)	1.61 (0.32)	0.81 (0.00)	0.16 (0.53)	0.15
K16554	4.18 (0.00)	-1.70 (0.02)	-0.14 (0.88)	2.88 (0.03)	nan (nan)	nan (nan)	nan
K20920	3.69 (0.00)	-1.29 (0.04)	0.23 (0.80)	2.41 (0.07)	nan (nan)	nan (nan)	nan
K20977	20.95 (0.99)	-1.38 (0.03)	0.01 (0.98)	3.39 (0.06)	0.58 (0.00)	0.62 (2.40e-05)	0.06
K20978	20.95 (0.99)	-1.38 (0.03)	0.01 (0.98)	3.39 (0.06)	0.58 (0.00)	0.62 (2.40e-05)	0.06
K20988	3.69 (0.00)	-1.29 (0.04)	0.23 (0.80)	2.41 (0.07)	nan (nan)	nan (nan)	nan

Supplementary Table 4: Results of regression analysis with and without phylogeny for limostatic classifer. For each feature, different models were applied: logistic regression (glm), logistic regression with phylogeny (phyloglm). [Because of the size of this table, it is not displayed in this document but available separately as a Supplementary Information file.]

Supplementary Table 5: Presence (+) or absence (-) of vps-related genes based on annotated genomes (accession numbers in Table S1) of all Vibrionaceae, including the (top) 11 species used to train the classifiers and the (bottom) four additional species used for prediction. Presence of *vps* genes refers to the presence of at least one of the *vpsN, vpsO, vpsM* genes. For *rbmC* genes, the predicted presence based on available genomes was corrected using the more specific annotation of Ref. [132].

Species	Category	*vps*	*rbmC*
Aliivibrio fischeri	Limostatic	+	-
Vibrio splendidus	Limostatic	-	-
Vibrio lentus	Limostatic	-	-
Vibrio tasmaniensis	Limostatic	-	-
Vibrio cyclitrophicus	Limostatic	-	-
Vibrio alginolyticus	Limostatic	+	-
Vibrio kanaloae	Limostatic	-	-
Vibrio sp. F10	Limostatic	+	-
Vibrio sp. ZF-129	Limokinetic	+	-
Vibrio anguillarum	Limokinetic	+	+*
Vibrio coralliilyticus	Limokinetic	+	+
Vibrio fortis	Limostatic	-	-
Vibrio campbellii	Limostatic	+	-
Vibrio furnissii	Limokinetic	+	+
Vibrio cholerae	Limokinetic	+	+

*Except strain FS-144.

References

[1] F. M. Lauro, D. McDougald, T. Thomas, T. J. Williams, S. Egan, S. Rice, M. Z. DeMaere, L. Ting, H. Ertan, J. Johnson, S. Ferriera, A. Lapidus, I. Anderson, N. Kyrpides, A. C. Munk, C. Detter, C. S. Han, M. V. Brown, F. T. Robb, S. Kjelleberg, and R. Cavicchioli, "The genomic basis of trophic strategy in marine bacteria," *PNAS*, vol. 106, pp. 15527–15533, Sept. 2009.

[2] J. P. Zehr, J. S. Weitz, and I. Joint, "How microbes survive in the open ocean," *Science*, vol. 357, pp. 646–647, Aug. 2017.

[3] S. Srinivasan and S. Kjelleberg, "Cycles of famine and feast: the starvation and outgrowth strategies of a marine Vibrio," *J. Biosci.*, vol. 23, pp. 501–511, Oct. 1998.

[4] M. Sebastián, M. Estrany, C. Ruiz-González, I. Forn, M. M. Sala, J. M. Gasol, and C. Marrasé, "High Growth Potential of Long-Term Starved Deep Ocean Opportunistic Heterotrophic Bacteria," *Front. Microbiol.*, vol. 10, p. 760, Apr. 2019.

[5] N. Jiao, T. Luo, Q. Chen, Z. Zhao, X. Xiao, J. Liu, Z. Jian, S. Xie, H. Thomas, G. J. Herndl, R. Benner, M. Gonsior, F. Chen, W.-J. Cai, and C. Robinson, "The microbial carbon pump and climate change," *Nat Rev Microbiol*, vol. 22, pp. 408–419, July 2024.

[6] M. A. Moran, E. B. Kujawinski, W. F. Schroer, S. A. Amin, N. R. Bates, E. M. Bertrand, R. Braakman, C. T. Brown, M. W. Covert, S. C. Doney, S. T. Dyhrman, A. S. Edison, A. M. Eren, N. M. Levine, L. Li, A. C. Ross, M. A. Saito, A. E. Santoro, D. Segrè, A. Shade, M. B. Sullivan, and A. Vardi, "Microbial metabolites in the marine carbon cycle," *Nat Microbiol*, vol. 7, pp. 508–523, Apr. 2022.

[7] M. Bergkessel, D. W. Basta, and D. K. Newman, "The physiology of growth arrest: uniting molecular and environmental microbiology," *Nat Rev Microbiol*, vol. 14, pp. 549–562, Sept. 2016.

[8] J. Dworkin and C. S. Harwood, "Metabolic Reprogramming and Longevity in Quiescence," *Annu. Rev. Microbiol.*, vol. 76, pp. 91–111, Sept. 2022.

[9] N. Wadhwa and H. C. Berg, "Bacterial motility: machinery and mechanisms," *Nat Rev Microbiol*, Sept. 2021.

[10] R. Stocker, "Marine Microbes See a Sea of Gradients," *Science*, vol. 338, pp. 628–633, Nov. 2012.

[11] J. M. Keegstra, F. Carrara, and R. Stocker, "The ecological roles of bacterial chemotaxis," *Nat Rev Microbiol*, Mar. 2022.

[12] N. I. Wisnoski and J. T. Lennon, "Scaling up and down: movement ecology for microorganisms," *Trends in Microbiology*, vol. 31, pp. 242–253, Mar. 2023.

[13] T. M. Hoehler and B. B. Jørgensen, "Microbial life under extreme energy limitation," *Nat Rev Microbiol*, vol. 11, pp. 83–94, Feb. 2013.

[14] B. Ni, R. Colin, H. Link, R. G. Endres, and V. Sourjik, "Growth-rate dependent resource investment in bacterial motile behavior quantitatively follows potential benefit of chemotaxis," *Proc. Natl. Acad. Sci. U.S.A.*, vol. 117, pp. 595–601, Jan. 2020.

[15] M. H. Larsen, N. Blackburn, J. L. Larsen, and J. E. Olsen, "Influences of temperature, salinity and starvation on the motility and chemotactic response of Vibrio anguillarum," *Microbiology*, vol. 150, pp. 1283–1290, May 2004.

[16] E. Yam and K. Tang, "Effects of starvation on aggregate colonization and motility of marine bacteria," *Aquat. Microb. Ecol.*, vol. 48, pp. 207–215, Aug. 2007.

[17] M. A. Lever, K. L. Rogers, K. G. Lloyd, J. Overmann, B. Schink, R. K. Thauer, T. M. Hoehler, and B. B. Jørgensen, "Life under extreme energy limitation: a synthesis of laboratory- and field-based investigations," *FEMS Microbiology Reviews*, vol. 39, pp. 688–728, Sept. 2015.

[18] X. Wei and W. D. Bauer, "Starvation-Induced Changes in Motility, Chemotaxis, and Flagellation of Rhizobium meliloti," *Appl. Environ. Microbiol.*, vol. 64, pp. 1708–1714, May 1998.

[19] K. Malmcrona-Friberg, A. Goodman, and S. Kjelleberg, "Chemotactic Responses of Marine *Vibrio* sp. Strain S14 (CCUG 15956) to Low-Molecular-Weight Substances under Starvation and Recovery Conditions," *Appl. Environ. Microbiol.*, vol. 56, pp. 3699–3704, Dec. 1990.

[20] X. Zhuang, S. Guo, Z. Li, Z. Zhao, S. Kojima, M. Homma, P. Wang, C. Lo, and F. Bai, "Live-cell fluorescence imaging reveals dynamic production and loss of bacterial flagella," *Mol Microbiol*, vol. 114, pp. 279–291, Aug. 2020.

[21] J. L. Ferreira, F. Z. Gao, F. M. Rossmann, A. Nans, S. Brenzinger, R. Hosseini, A. Wilson, A. Briegel, K. M. Thormann, P. B. Rosenthal, and M. Beeby, "Gammaproteobacteria eject their polar flagella under nutrient depletion, retaining flagellar motor relic structures," *PLOS Biology*, vol. 17, p. e3000165, Mar. 2019.

[22] S. Stretton, S. J. Danon, S. Kjelleberg, and A. E. Goodman, "Changes in cell morphology and motility in the marine Vibrio sp. strain S14 during conditions of starvation and recovery," *FEMS Microbiology Letters*, vol. 146, pp. 23–29, Jan. 2006.

[23] J.-B. Raina, B. S. Lambert, D. H. Parks, C. Rinke, N. Siboni, A. Bramucci, M. Ostrowski, B. Signal, A. Lutz, H. Mendis, F. Rubino, V. I. Fernandez, R. Stocker, P. Hugenholtz, G. W. Tyson, and J. R. Seymour, "Chemotaxis shapes the microscale organization of the ocean's microbiome," *Nature*, vol. 605, pp. 132–138, May 2022.

[24] U. Alcolombri, F. J. Peaudecerf, V. I. Fernandez, L. Behrendt, K. S. Lee, and R. Stocker, "Sinking enhances the degradation of organic particles by marine bacteria," *Nat. Geosci.*, vol. 14, pp. 775–780, Sept. 2021.

[25] T. Kiørboe, *A mechanistic approach to plankton ecology*. Princeton University Press, 2008.

[26] B. S. Lambert, V. I. Fernandez, and R. Stocker, "Motility drives bacterial encounter with particles responsible for carbon export throughout the ocean," *Limnol. Oceanogr.*, vol. 4, pp. 113–118, Oct. 2019.

[27] J. Słomka, U. Alcolombri, E. Secchi, R. Stocker, and V. I. Fernandez, "Encounter rates between bacteria and small sinking particles," *New J. Phys.*, vol. 22, p. 043016, Apr. 2020.

[28] B. Borer, I. H. Zhang, A. E. Baker, G. A. O'Toole, and A. R. Babbin, "Porous marine snow differentially benefits chemotactic, motile, and nonmotile bacteria," *PNAS Nexus*, vol. 2, p. pgac311, Feb. 2023.

[29] M. S. Datta, E. Sliwerska, J. Gore, M. F. Polz, and O. X. Cordero, "Microbial interactions lead to rapid micro-scale successions on model marine particles," *Nature Communications*, vol. 7, p. 11965, June 2016.

[30] T. M. Steinum, S. Karataş, N. T. Martinussen, P. M. Meirelles, F. L. Thompson, and D. J. Colquhoun, "Multilocus Sequence Analysis of Close Relatives Vibrio anguillarum and Vibrio ordalii," *Appl Environ Microbiol*, vol. 82, pp. 5496–5504, Sept. 2016.

[31] E. Leifson, B. J. Cosenza, R. Miuirichelano, and I. C. Cleverdon, "Motile marine bacteria. Techniques, ecology and general characteristics," *Journal of Bacteriology*, vol. 87, pp. 652–666, 1964.

[32] K. Son, J. S. Guasto, and R. Stocker, "Bacteria can exploit a flagellar buckling instability to change direction," *Nature Physics*, vol. 9, pp. 494–498, Aug. 2013.

[33] L. Xie, T. Altindal, S. Chattopadhyay, and X.-L. Wu, "Bacterial flagellum as a propeller and as a rudder for efficient chemotaxis," *Proc. Natl. Acad. Sci. U.S.A.*, vol. 108, pp. 2246–2251, Feb. 2011.

[34] T. T. Renault, A. O. Abraham, T. Bergmiller, G. Paradis, S. Rainville, E. Charpentier, C. C. Guet, Y. Tu, K. Namba, J. P. Keener, T. Minamino, and M. Erhardt, "Bacterial flagella grow through an injection-diffusion mechanism," *eLife*, vol. 6, p. e23136, Mar. 2017.

[35] L. Turner, A. S. Stern, and H. C. Berg, "Growth of Flagellar Filaments of Escherichia coli Is Independent of Filament Length," *Journal of Bacteriology*, vol. 194, pp. 2437–2442, May 2012.

[36] X.-Y. Zhuang and C.-J. Lo, "Construction and Loss of Bacterial Flagellar Filaments," *Biomolecules*, vol. 10, p. 1528, Nov. 2020.

[37] M. Chen, Z. Zhao, J. Yang, K. Peng, M. A. Baker, F. Bai, and C.-J. Lo, “Length-dependent flagellar growth of Vibrio alginolyticus revealed by real time fluorescent imaging,” *eLife*, vol. 6, p. e22140, Jan. 2017.

[38] S. Chattopadhyay, R. Moldovan, C. Yeung, and X. L. Wu, “Swimming efficiency of bacterium *Escherichia coli*,” *Proceedings of the National Academy of Sciences*, vol. 103, pp. 13712–13717, Sept. 2006.

[39] J. R. Taylor and R. Stocker, “Trade-Offs of Chemotactic Foraging in Turbulent Water,” *Science*, vol. 338, pp. 675–679, Nov. 2012.

[40] E. Biselli, S. J. Schink, and U. Gerland, “Slower growth of *Escherichia coli* leads to longer survival in carbon starvation due to a decrease in the maintenance rate,” *Mol Syst Biol*, vol. 16, June 2020.

[41] C. P. Kempes, P. M. van Bodegom, D. Wolpert, E. Libby, J. Amend, and T. Hoehler, “Drivers of Bacterial Maintenance and Minimal Energy Requirements,” *Front. Microbiol.*, vol. 8, Jan. 2017.

[42] P. S. Amy and R. Y. Morita, “Starvation-Survival Patterns of Sixteen Freshly Isolated Open-Ocean Bacteria,” *Appl. Environ. Microbiol.*, vol. 45, pp. 1109–1115, Mar. 1983.

[43] M. Sebastián, J. Auguet, C. X. Restrepo-Ortiz, M. M. Sala, C. Marrasé, and J. M. Gasol, “Deep ocean prokaryotic communities are remarkably malleable when facing long-term starvation,” *Environmental Microbiology*, vol. 20, pp. 713–723, Feb. 2018.

[44] J. G. Mitchell, “The influence of cell size on marine bacterial motility and energetics,” *Microb Ecol*, vol. 22, pp. 227–238, Dec. 1991.

[45] B. R. K. Roller, C. Hellerschmied, Y. Wu, T. P. Miettinen, A. L. Gomez, S. R. Manalis, and M. F. Polz, “Single-cell mass distributions reveal simple rules for achieving steady-state growth,” *mBio*, vol. 14, pp. e01585–23, Oct. 2023.

[46] Z. Monemhaghdoust, F. Montfort, Y. Emery, C. Depeursinge, and C. Moser, “Off-axis digital holographic camera for quantitative phase microscopy,” *Biomed. Opt. Express*, vol. 5, p. 1721, June 2014.

[47] E. R. Oldewurtel, Y. Kitahara, and S. Van Teeffelen, “Robust surface-to-mass coupling and turgor-dependent cell width determine bacterial dry-mass density,” *Proc. Natl. Acad. Sci. U.S.A.*, vol. 118, p. e2021416118, Aug. 2021.

[48] N. Cermak, J. W. Becker, S. M. Knudsen, S. W. Chisholm, S. R. Manalis, and M. F. Polz, “Direct single-cell biomass estimates for marine bacteria via Archimedes’ principle,” *ISME J*, vol. 11, pp. 825–828, Mar. 2017.

[49] W. R. Shoemaker, S. E. Jones, M. E. Muscarella, M. G. Behringer, B. K. Lehmkuhl, and J. T. Lennon, “Microbial population dynamics and evolutionary outcomes under extreme energy limitation,” *Proc. Natl. Acad. Sci. U.S.A.*, vol. 118, p. e2101691118, Aug. 2021.

[50] S. J. Schink, E. Biselli, C. Ammar, and U. Gerland, “Death Rate of E. coli during Starvation Is Set by Maintenance Cost and Biomass Recycling,” *Cell Systems*, vol. 9, pp. 64–73.e3, July 2019.

[51] L. Paoli, H.-J. Ruscheweyh, C. C. Forneris, F. Hubrich, S. Kautsar, A. Bhushan, A. Lotti, Q. Clayssen, G. Salazar, A. Milanese, C. I. Carlström, C. Papadopoulou, D. Gehrig, M. Karasikov, H. Mustafa, M. Larralde, L. M. Carroll, P. Sánchez, A. A. Zayed, D. R. Cronin, S. G. Acinas, P. Bork, C. Bowler, T. O. Delmont, J. M. Gasol, A. D. Gossert, A. Kahles, M. B. Sullivan, P. Wincker, G. Zeller, S. L. Robinson, J. Piel, and S. Sunagawa, “Biosynthetic potential of the global ocean microbiome,” *Nature*, vol. 607, pp. 111–118, July 2022.

[52] F.-Q. Wang, D. Bartosik, C. Sidhu, R. Siebers, D.-C. Lu, A. Trautwein-Schult, D. Becher, B. Huettel, J. Rick, I. V. Kirstein, K. H. Wiltshire, T. Schweder, B. M. Fuchs, M. M. Bengtsson, H. Teeling, and R. I. Amann, “Particle-attached bacteria act as gatekeepers in the decomposition of complex phytoplankton polysaccharides,” *Microbiome*, vol. 12, p. 32, Feb. 2024.

[53] F. Azam and F. Malfatti, “Microbial structuring of marine ecosystems,” *Nature Reviews Microbiology*, vol. 5, pp. 782–791, Oct. 2007.

[54] T. T. H. Nguyen, E. J. Zakem, A. Ebrahimi, J. Schwartzman, T. Caglar, K. Amarnath, U. Alcolombri, F. J. Peaudecerf, T. Hwa, R. Stocker, O. X. Cordero, and N. M. Levine, "Microbes contribute to setting the ocean carbon flux by altering the fate of sinking particulates," *Nat Commun*, vol. 13, p. 1657, Dec. 2022.

[55] R. Colin, B. Ni, L. Laganenka, and V. Sourjik, "Multiple functions of flagellar motility and chemotaxis in bacterial physiology," *FEMS Microbiology Reviews*, vol. 45, p. fuab038, July 2021.

[56] C. D. Amsler, M. Cho, and P. Matsumura, "Multiple factors underlying the maximum motility of *Escherichia coli* as cultures enter post-exponential growth.," *J. Bacteriol.*, vol. 175, pp. 6238–6244, Oct. 1993.

[57] J. G. Mitchell, L. Pearson, A. Bonazinga, S. Dillon, H. Khouri, and R. Paxinos, "Long lag times and high velocities in the motility of natural assemblages of marine bacteria.," *Appl. Environ. Microbiol.*, vol. 61, no. 3, pp. 877–882, 1995.

[58] T. Honda, J. Cremer, L. Mancini, Z. Zhang, T. Pilizota, and T. Hwa, "Coordination of gene expression with cell size enables *Escherichia coli* to efficiently maintain motility across conditions," *Proc. Natl. Acad. Sci. U.S.A.*, vol. 119, p. e2110342119, Sept. 2022.

[59] J. Cremer, T. Honda, Y. Tang, J. Wong-Ng, M. Vergassola, and T. Hwa, "Chemotaxis as a navigation strategy to boost range expansion," *Nature*, vol. 575, pp. 658–663, Nov. 2019.

[60] S. Gude, E. Pinçe, K. M. Taute, A.-B. Seinen, T. S. Shimizu, and S. J. Tans, "Bacterial coexistence driven by motility and spatial competition," *Nature*, vol. 578, pp. 588–592, Feb. 2020.

[61] T. Fenchel, "Eppur si muove: many water column bacteria are motile," *Aquatic Microbial Ecology*, vol. 24, pp. 197–201, 2001.

[62] H. Grossart, L. Riemann, and F. Azam, "Bacterial motility in the sea and its ecological implications," *Aquat. Microb. Ecol.*, vol. 25, pp. 247–258, 2001.

[63] S. Zhu and B. Gao, "Bacterial Flagella Loss under Starvation," *Trends in Microbiology*, vol. 28, pp. 785–788, Oct. 2020.

[64] J. Pernthaler, "Predation on prokaryotes in the water column and its ecological implications," *Nat Rev Microbiol*, vol. 3, pp. 537–546, July 2005.

[65] C. Matz and K. Jürgens, "High Motility Reduces Grazing Mortality of Planktonic Bacteria," *Appl. Environ. Microbiol.*, vol. 71, pp. 921–929, Feb. 2005.

[66] F. de Schaetzen, M. Fan, U. Alcolombri, F. J. Peaudecerf, D. Drissner, M. J. Loessner, R. Stocker, and M. Schuppler, "Random encounters and amoeba locomotion drive the predation of *Listeria monocytogenes* by *Acanthamoeba castellanii*," *Proc. Natl. Acad. Sci. U.S.A.*, vol. 119, p. e2122659119, Aug. 2022.

[67] Y.-W. Lien, D. Amendola, K. S. Lee, N. Bartlau, J. Xu, G. Furusawa, M. F. Polz, R. Stocker, G. L. Weiss, and M. Pilhofer, "Mechanism of bacterial predation via ixotrophy," *Science*, vol. 386, p. eadp0614, Oct. 2024.

[68] S. Z. Schade, J. Adler, and H. Ris, "How Bacteriophage x Attacks Motile Bacteria," *Journal of Virology*, vol. 1, no. 3, pp. 599–609, 1967.

[69] J. Y. Yen, K. M. Broadway, and B. E. Scharf, "Minimum Requirements of Flagellation and Motility for Infection of Agrobacterium sp. Strain H13-3 by Flagellotropic Bacteriophage 7-7-1," *Appl. Environ. Microbiol.*, vol. 78, pp. 7216–7222, Oct. 2012.

[70] D. E. Hunt, L. A. David, D. Gevers, S. P. Preheim, E. J. Alm, and M. F. Polz, "Resource Partitioning and Sympatric Differentiation Among Closely Related Bacterioplankton," *Science*, vol. 320, pp. 1081–1085, May 2008.

[71] O. X. Cordero, H. Wildschutte, B. Kirkup, S. Proehl, L. Ngo, F. Hussain, F. Le Roux, T. Mincer, and M. F. Polz, "Ecological Populations of Bacteria Act as Socially Cohesive Units of Antibiotic Production and Resistance," *Science*, vol. 337, pp. 1228–1231, Sept. 2012.

[72] Y. Yawata, O. X. Cordero, F. Menolascina, J.-H. Hehemann, M. F. Polz, and R. Stocker, “Competition-dispersal tradeoff ecologically differentiates recently speciated marine bacterioplankton populations,” *Proc. Natl. Acad. Sci. U.S.A*, vol. 111, pp. 5622–5627, Apr. 2014.

[73] J.-H. Hehemann, P. Arevalo, M. S. Datta, X. Yu, C. H. Corzett, A. Henschel, S. P. Preheim, S. Timberlake, E. J. Alm, and M. F. Polz, “Adaptive radiation by waves of gene transfer leads to fine-scale resource partitioning in marine microbes,” *Nature Communications*, vol. 7, p. 12860, Sept. 2016.

[74] Y. Ben-Haim and E. Rosenberg, “A novel Vibrio sp. pathogen of the coral Pocillopora damicornis,” *Marine Biology*, vol. 141, pp. 47–55, July 2002.

[75] R. M. Welsh, J. R. Zaneveld, S. M. Rosales, J. P. Payet, D. E. Burkepile, and R. V. Thurber, “Bacterial predation in a marine host-associated microbiome,” *ISME J*, vol. 10, pp. 1540–1544, June 2016.

[76] R. Stocker, J. R. Seymour, A. Samadani, D. E. Hunt, and M. F. Polz, “Rapid chemotactic response enables marine bacteria to exploit ephemeral microscale nutrient patches,” *Proc. Natl. Acad. Sci. U.S.A*, vol. 105, pp. 4209–4214, Mar. 2008.

[77] E. C. Kaeppel, A. Gärdes, S. Seebah, H.-P. Grossart, and M. S. Ullrich, “*Marinobacter adhaerens sp. nov.*, isolated from marine aggregates formed with the diatom Thalassiosira weissflogii,” *International Journal of Systematic and Evolutionary Microbiology*, vol. 62, pp. 124–128, Jan. 2012.

[78] G. Loy and A. Zelinsky, “A Fast Radial Symmetry Transform for Detecting Points of Interest,” *Computer Vision ECCV 2002*, vol. 2350, pp. 358–368, 2002. Series Title: Lecture Notes in Computer Science.

[79] D. B. Allen, T. Caswell, N. C. Keim, C. M. van der Wel, and R. W. Verweij, “Trackpy,” 2021. 10.5281/zenodo.4682814.

[80] E. E. Clerc and et al., “Strong chemotaxis by marine bacteria towards polysaccharides is enhanced by the abundant organosulfur compound DMSP,” *Nature Communications*, vol. 14, p. 8080, 2023.

[81] A. Savitzky and M. J. E. Golay, “Smoothing and Differentiation of Data by Simplified Least Squares Procedures.,” *Anal. Chem.*, vol. 36, pp. 1627–1639, July 1964.

[82] F. Asnicar, A. M. Thomas, F. Beghini, C. Mengoni, S. Manara, P. Manghi, Q. Zhu, M. Bolzan, F. Cumbo, U. May, J. G. Sanders, M. Zolfo, E. Kopylova, E. Pasolli, R. Knight, S. Mirarab, C. Huttenhower, and N. Segata, “Precise phylogenetic analysis of microbial isolates and genomes from metagenomes using PhyloPhlAn 3.0,” *Nat Commun*, vol. 11, p. 2500, May 2020.

[83] M. Streichan, J. R. Golecki, and G. SchÃ¶n, “Polyphosphate-accumulating bacteria from sewage plants with different proceses for biological phosphorus removal,” *FEMS Microbiology Letters*, vol. 73, pp. 113–124, Feb. 1990.

[84] D. P. Mesquita, A. L. Amaral, C. Leal, M. Carvalheira, J. R. Cunha, A. Oehmen, M. A. M. Reis, and E. C. Ferreira, “Monitoring intracellular polyphosphate accumulation in enhanced biological phosphorus removal systems by quantitative image analysis,” *Water Science and Technology*, vol. 69, pp. 2315–2323, June 2014.

[85] J. Kacmar, R. Carlson, S. J. Balogh, and F. Srienc, “Staining and quantification of poly-3-hydroxybutyrate in *Saccharomyces cerevisiae* and *Cupriavidus necator* B cell populations using automated flow cytometry,” *Cytometry*, vol. 69A, pp. 27–35, Jan. 2006.

[86] S. Berg, D. Kutra, T. Kroeger, C. N. Straehle, B. X. Kausler, C. Haubold, M. Schiegg, J. Ales, T. Beier, M. Rudy, K. Eren, J. I. Cervantes, B. Xu, F. Beuttenmueller, A. Wolny, C. Zhang, U. Koethe, F. A. Hamprecht, and A. Kreshuk, “ilastik: interactive machine learning for (bio)image analysis,” *Nat Methods*, vol. 16, pp. 1226–1232, Dec. 2019.

[87] A. Hernández-Plaza, D. Szklarczyk, J. Botas, C. Cantalapiedra, J. Giner-Lamia, D. R. Mende, R. Kirsch, T. Rattei, I. Letunic, L. Jensen, P. Bork, C. von Mering, and J. Huerta-Cepas, “eggNOG 6.0: enabling comparative genomics across 12 535 organisms,” *Nucleic Acids Research*, vol. 51, pp. D389–D394, Jan. 2023.

[88] M. Kanehisa and S. Goto, "KEGG: Kyoto Encyclopedia of Genes and Genomes," *Nucleic Acids Research*, vol. 28, No. 1, pp. 27–30, 2000.

[89] M. Kuhn, "Building Predictive Models in R Using the caret Package," *J. Stat. Soft.*, vol. 28, pp. 1 – 26, Nov. 2008. 10.18637/jss.v028.i05.

[90] L. S. Tung Ho and C. Ané, "A Linear-Time Algorithm for Gaussian and Non-Gaussian Trait Evolution Models," *Systematic Biology*, vol. 63, pp. 397–408, May 2014.

[91] B. Q. Minh, H. A. Schmidt, O. Chernomor, D. Schrempf, M. D. Woodhams, A. Von Haeseler, and R. Lanfear, "IQ-TREE 2: New Models and Efficient Methods for Phylogenetic Inference in the Genomic Era," *Molecular Biology and Evolution*, vol. 37, pp. 1530–1534, May 2020.

[92] T. S. Shimizu, N. Delalez, K. Pichler, and H. C. Berg, "Monitoring bacterial chemotaxis by using bioluminescence resonance energy transfer: Absence of feedback from the flagellar motors," *Proceedings of the National Academy of Sciences*, vol. 103, pp. 2093–2097, Feb. 2006.

[93] L. Gómez-Consarnau, N. Akram, K. Lindell, A. Pedersen, R. Neutze, D. L. Milton, J. M. González, and J. Pinhassi, "Proteorhodopsin Phototrophy Promotes Survival of Marine Bacteria during Starvation," *PLoS Biol*, vol. 8, p. e1000358, Apr. 2010.

[94] L. Steindler, M. S. Schwalbach, D. P. Smith, F. Chan, and S. J. Giovannoni, "Energy Starved Candidatus Pelagibacter Ubique Substitutes Light-Mediated ATP Production for Endogenous Carbon Respiration," *PLoS ONE*, vol. 6, p. e19725, May 2011.

[95] R. Bar-Shalom, A. Rozenberg, M. Lahyani, B. Hassanzadeh, G. Sahoo, M. Haber, I. Burgsdorf, X. Tang, V. Squatrito, L. Gomez-Consarnau, O. Béjà, and L. Steindler, "Rhodopsin-mediated nutrient uptake by cultivated photoheterotrophic *Verrucomicrobiota*," *The ISME Journal*, vol. 17, pp. 1063–1073, July 2023.

[96] J. M. Walter, D. Greenfield, C. Bustamante, and J. Liphardt, "Light-powering Escherichia coli with proteorhodopsin," *Proceedings of the National Academy of Sciences*, vol. 104, pp. 2408–2412, Feb. 2007.

[97] K. Inoue, H. Ono, R. Abe-Yoshizumi, S. Yoshizawa, H. Ito, K. Kogure, and H. Kandori, "A light-driven sodium ion pump in marine bacteria," *Nat Commun*, vol. 4, p. 1678, Apr. 2013.

[98] D. Kadouri, E. Jurkevitch, and Y. Okon, "Involvement of the Reserve Material Poly-Hydroxybutyrate in *Azospirillum brasilense* Stress Endurance and Root Colonization," *Applied and Environmental Microbiology*, vol. 69, p. 7, 2003.

[99] M. H. Rashid, N. N. Rao, and A. Kornberg, "Inorganic Polyphosphate Is Required for Motility of Bacterial Pathogens," *J Bacteriol*, vol. 182, pp. 225–227, Jan. 2000.

[100] K. Sekar, S. M. Linker, J. Nguyen, A. Grünhagen, R. Stocker, and U. Sauer, "Bacterial Glycogen Provides Short-Term Benefits in Changing Environments," *Appl. Environ. Microbiol.*, vol. 86, pp. e00049–20, Apr. 2020.

[101] L. Bourassa and A. Camilli, "Glycogen contributes to the environmental persistence and transmission of *Vibrio cholerae* ," *Molecular Microbiology*, vol. 72, pp. 124–138, Mar. 2009.

[102] N. J. Watmough and F. E. Frerman, "The electron transfer flavoprotein: Ubiquinone oxidoreductases," *Biochimica et Biophysica Acta (BBA) - Bioenergetics*, vol. 1797, pp. 1910–1916, Dec. 2010.

[103] J. A. Imlay, "The molecular mechanisms and physiological consequences of oxidative stress: lessons from a model bacterium," *Nat Rev Microbiol*, vol. 11, pp. 443–454, July 2013.

[104] F. Baltar, T. Reinthaler, G. J. Herndl, and J. Pinhassi, "Major Effect of Hydrogen Peroxide on Bacterioplankton Metabolism in the Northeast Atlantic," *PLoS ONE*, vol. 8, p. e61051, Apr. 2013.

[105] J. J. Morris, A. L. Rose, and Z. Lu, "Reactive oxygen species in the world ocean and their impacts on marine ecosystems," *Redox Biology*, vol. 52, p. 102285, June 2022.

[106] D. McDougald, L. Gong, S. Srinivasan, E. Hild, L. Thompson, S. A. Rice, and S. Kjelleberg, "Defences against oxidative stress during starvation in bacteria," *Van Leeuwenhoek*, 2002.

[107] M. Ostrowski, R. Cavicchioli, M. Blaauw, and J. C. Gottschal, "Specific Growth Rate Plays a Critical Role in Hydrogen Peroxide Resistance of the Marine Oligotrophic Ultramicrobacterium *Sphingomonas alaskensis* Strain RB2256," *Appl Environ Microbiol*, vol. 67, pp. 1292–1299, Mar. 2001.

[108] S. Cesar, L. Willis, and K. C. Huang, "Bacterial respiration during stationary phase induces intracellular damage that leads to delayed regrowth," *iScience*, vol. 25, p. 103765, Mar. 2022.

[109] L. Calhoun and Y. Kwon, "Structure, function and regulation of the DNA-binding protein Dps and its role in acid and oxidative stress resistance in Escherichia coli: a review: Escherichia coli Dps protein," *Journal of Applied Microbiology*, vol. 110, pp. 375–386, Feb. 2011.

[110] E. Chiancone and P. Ceci, "The multifaceted capacity of Dps proteins to combat bacterial stress conditions: Detoxification of iron and hydrogen peroxide and DNA binding," *Biochimica et Biophysica Acta (BBA) - General Subjects*, vol. 1800, pp. 798–805, Aug. 2010.

[111] A. Martinez and R. Kolter, "Protection of DNA during oxidative stress by the nonspecific DNA-binding protein Dps," *J Bacteriol*, vol. 179, pp. 5188–5194, Aug. 1997.

[112] A. Klingner, A. Bartsch, M. Dogs, I. Wagner-Döbler, D. Jahn, M. Simon, T. Brinkhoff, J. Becker, and C. Wittmann, "Large-Scale ^{13} C Flux Profiling Reveals Conservation of the Entner-Doudoroff Pathway as a Glycolytic Strategy among Marine Bacteria That Use Glucose," *Appl Environ Microbiol*, vol. 81, pp. 2408–2422, Apr. 2015.

[113] J. A. Imlay, "Where in the world do bacteria experience oxidative stress?: Oxidative stress in natural environments," *Environ Microbiol*, vol. 21, pp. 521–530, Feb. 2019.

[114] J. J. Morris, Z. I. Johnson, M. J. Szul, M. Keller, and E. R. Zinser, "Dependence of the Cyanobacterium Prochlorococcus on Hydrogen Peroxide Scavenging Microbes for Growth at the Ocean's Surface," *PLoS ONE*, vol. 6, p. e16805, Feb. 2011.

[115] J. J. Morris, R. E. Lenski, and E. R. Zinser, "The Black Queen Hypothesis: Evolution of Dependencies through Adaptive Gene Loss," *mBio*, vol. 3, pp. e00036–12, May 2012.

[116] M. P. Ferla and W. M. Patrick, "Bacterial methionine biosynthesis," *Microbiology*, vol. 160, pp. 1571–1584, Aug. 2014.

[117] B. S. Goldman and J. R. Roth, "Genetic structure and regulation of the cysG gene in Salmonella typhimurium," *J Bacteriol*, vol. 175, pp. 1457–1466, Mar. 1993.

[118] B. C. Tripathy, I. Sherameti, and R. Oelmüller, "Siroheme: An essential component for life on earth," *Plant Signaling & Behavior*, vol. 5, pp. 14–20, Jan. 2010.

[119] E. M. Bertrand, D. M. Moran, M. R. McIlvin, J. M. Hoffman, A. E. Allen, and M. A. Saito, "Methionine synthase interreplacement in diatom cultures and communities: Implications for the persistence of B $_{12}$ use by eukaryotic phytoplankton," *Limnol. Oceanogr.*, vol. 58, pp. 1431–1450, July 2013.

[120] B. Ezraty, A. Gennaris, F. Barras, and J.-F. Collet, "Oxidative stress, protein damage and repair in bacteria," *Nat Rev Microbiol*, vol. 15, pp. 385–396, July 2017.

[121] V. Sperandio, A. G. Torres, and J. B. Kaper, "Quorum sensing *Escherichia coli* regulators B and C (QseBC): a novel two-component regulatory system involved in the regulation of flagella and motility by quorum sensing in *E. coli*: QseBC regulates flagella and motility in *E. coli*," *Molecular Microbiology*, vol. 43, pp. 809–821, Feb. 2002.

[122] J.-L. Hsu, H.-C. Chen, H.-L. Peng, and H.-Y. Chang, "Characterization of the Histidine-containing Phosphotransfer Protein B-mediated Multistep Phosphorelay System in Pseudomonas aeruginosa PAO1," *Journal of Biological Chemistry*, vol. 283, pp. 9933–9944, Apr. 2008.

[123] C. V. Rao, G. D. Glekas, and G. W. Ordal, "The three adaptation systems of Bacillus subtilis chemotaxis," *Trends in Microbiology*, vol. 16, pp. 480–487, Oct. 2008.

[124] G. Lan, P. Sartori, S. Neumann, V. Sourjik, and Y. Tu, “The energy–speed–accuracy trade-off in sensory adaptation,” *Nature Physics*, vol. 8, pp. 422–428, May 2012.

[125] K. Stoderegger and G. Herndl, “Production of exopolymer particles by marine bacterioplankton under contrasting turbulence conditions,” *Mar. Ecol. Prog. Ser.*, vol. 189, pp. 9–16, 1999.

[126] A. Buffet, E. P. C. Rocha, and O. Rendueles, “Nutrient conditions are primary drivers of bacterial capsule maintenance in Klebsiella,” 2021.

[127] C. Schwechheimer, K. Hebert, S. Tripathi, P. K. Singh, K. A. Floyd, E. R. Brown, M. E. Porcella, J. Osorio, J. T. M. Kiblen, F. A. Pagliai, K. Drescher, S. M. Rubin, and F. H. Yildiz, “A tyrosine phosphoregulatory system controls exopolysaccharide biosynthesis and biofilm formation in Vibrio cholerae,” *PLoS Pathog*, vol. 16, p. e1008745, Aug. 2020.

[128] J. C. N. Fong, K. A. Syed, K. E. Klose, and F. H. Yildiz, “Role of Vibrio polysaccharide (vps) genes in VPS production, biofilm formation and Vibrio cholerae pathogenesis,” *Microbiology*, vol. 156, pp. 2757–2769, Sept. 2010.

[129] D. L. Gibson, A. P. White, S. D. Snyder, S. Martin, C. Heiss, P. Azadi, M. Surette, and W. W. Kay, “*Salmonella* Produces an O-Antigen Capsule Regulated by AgfD and Important for Environmental Persistence,” *J Bacteriol*, vol. 188, pp. 7722–7730, Nov. 2006.

[130] Y. Zheng, H. Wang, L. Huang, T. Zhang, B. Zong, X. Ren, Y. Zhu, F. Song, X. Wang, H. Chen, and C. Tan, “Effect of O antigen ligase gene mutation on oxidative stress resistance and pathogenicity of NMEC strain RS218,” *Microbial Pathogenesis*, vol. 136, p. 103656, Nov. 2019.

[131] F. H. Yildiz and K. L. Visick, “Vibrio biofilms: so much the same yet so different,” *Trends in Microbiology*, vol. 17, pp. 109–118, Mar. 2009.

[132] X. Huang, T. Nero, R. Weerasekera, K. H. Matej, A. Hinbest, Z. Jiang, R. F. Lee, L. Wu, C. Chak, J. Nijjer, I. Gibaldi, H. Yang, N. Gamble, W.-L. Ng, S. A. Malaker, K. Sumigray, R. Olson, and J. Yan, “Vibrio cholerae biofilms use modular adhesins with glycan-targeting and nonspecific surface binding domains for colonization,” *Nat Commun*, vol. 14, p. 2104, Apr. 2023.

[133] M. A. Matilla and T. Krell, “The effect of bacterial chemotaxis on host infection and pathogenicity,” *FEMS Microbiology Reviews*, vol. 42, Jan. 2018.

[134] J.-B. Raina, V. Fernandez, B. Lambert, R. Stocker, and J. R. Seymour, “The role of microbial motility and chemotaxis in symbiosis,” *Nature Reviews Microbiology*, vol. 17, pp. 284–294, May 2019.

[135] F. D. Bushman, K. McCormick, and S. Sherrill-Mix, “Virus structures constrain transmission modes,” *Nat Microbiol*, vol. 4, pp. 1778–1780, July 2019.

[136] M. Pagel, “Detecting Correlated Evolution on Phylogenies: A General Method for the Comparative Analysis of Discrete Characters,” *Proceedings: Biological Sciences*, vol. 255, no. 1342, pp. 37–45, 1994.

[137] L. J. Revell, “phytools 2.0: an updated R ecosystem for phylogenetic comparative methods (and other things),” *PeerJ*, vol. 12, p. e16505, Jan. 2024.

[138] M. Bressac, E. C. Laurenceau-Cornec, F. Kennedy, A. E. Santoro, N. L. Paul, N. Briggs, F. Carvalho, and P. W. Boyd, “Decoding drivers of carbon flux attenuation in the oceanic biological pump,” *Nature*, vol. 633, pp. 587–593, Sept. 2024.

[139] T. N. Enke, M. S. Datta, J. Schwartzman, N. Cermak, D. Schmitz, J. Barrere, A. Pascual-García, and O. X. Cordero, “Modular Assembly of Polysaccharide-Degrading Marine Microbial Communities,” *Current Biology*, vol. 29, pp. 1528–1535.e6, May 2019.

[140] G. D’Souza, A. Ebrahimi, A. Stubbusch, M. Daniels, J. Keegstra, R. Stocker, O. Cordero, and M. Ackermann, “Cell aggregation is associated with enzyme secretion strategies in marine polysaccharide-degrading bacteria,” *ISME J*, Feb. 2023.

[141] R. E. Szabo, S. Pontrelli, J. Grilli, J. A. Schwartzman, S. Pollak, U. Sauer, and O. X. Cordero, "Historical contingencies and phage induction diversify bacterioplankton communities at the microscale," *Proc. Natl. Acad. Sci. U.S.A.*, vol. 119, p. e2117748119, July 2022.

[142] M. R. Lewis, D. Hebert, W. G. Harrison, T. Platt, and N. S. Oakey, "Vertical Nitrate Fluxes in the Oligotrophic Ocean," *Science*, vol. 234, pp. 870–873, Nov. 1986.

[143] R. Keil and D. Kirchman, "Utilization of dissolved protein and amino acids in the northern Sargasso Sea," *Aquat. Microb. Ecol.*, vol. 18, pp. 293–300, 1999.

[144] N. H. Borch and D. L. Kirchman, "Concentration and composition of dissolved combined neutral sugars (polysaccharides) in seawater determined by HPLC-PAD," *Marine Chemistry*, vol. 57, pp. 85–95, May 1997.

[145] M. Zampieri, M. Hörl, F. Hotz, N. F. Müller, and U. Sauer, "Regulatory mechanisms underlying coordination of amino acid and glucose catabolism in Escherichia coli," *Nat Commun*, vol. 10, p. 3354, Dec. 2019.

[146] D. Schellenberg and E. Furlongs, "Resolution of the Multiplicity of the Glutamate and Aspartate Transport Systems of Escherichia coli," *Journal of Biological Chemistry*, vol. 252, no. 24, pp. 9055–9064, 1977.

[147] B. Ayo, M. Unanue, I. Azúa, G. Gorsky, C. Turley, and J. Iriberri, "Kinetics of glucose and amino acid uptake by attached and free-living marine bacteria in oligotrophic waters," *Marine Biology*, vol. 138, pp. 1071–1076, May 2001.

[148] C. Kaleta, S. Schäuble, U. Rinas, and S. Schuster, "Metabolic costs of amino acid and protein production in *Escherichia coli*," *Biotechnology Journal*, vol. 8, pp. 1105–1114, Sept. 2013.

[149] J. Davis and R. Benner, "Quantitative estimates of labile and semi-labile dissolved organic carbon in the western Arctic Ocean: A molecular approach," *Limnol. Oceanogr*, vol. 52(6), no. 6, pp. 2434–2444, 2007.

[150] G. G. Geesey and R. Y. Morita, "Capture of Arginine at Low Concentrations by a Marine Psychrophilic Bacterium," *Appl. Environ. Microbiol.*, vol. 38, pp. 1092–1097, Dec. 1979.

[151] N. Norris, N. M. Levine, V. I. Fernandez, and R. Stocker, "Mechanistic model of nutrient uptake explains dichotomy between marine oligotrophic and copiotrophic bacteria," *PLoS Comput Biol*, vol. 17, p. e1009023, May 2021.

[152] M. Picheral, S. Searson, V. Taillandier, A. Bricaud, E. Boss, L. Stemmann, G. Gorsky, C. T. O. Consortium, and P. T. O. Expedition, "Vertical profiles of environmental parameters measured from physical, optical and imaging sensors during Tara Oceans expedition 2009-2013," 2014. Type: data set.

[153] S. Chandrasekhar, "Stochastic Problems in Physics and Astronomy," *Rev. Mod. Phys.*, vol. 15, pp. 1–89, Jan. 1943.

[154] J. Taktikos, H. Stark, and V. Zaburdaev, "How the Motility Pattern of Bacteria Affects Their Dispersal and Chemotaxis," *PLoS ONE*, vol. 8, p. e81936, Dec. 2013.

[155] K. Son, F. Menolascina, and R. Stocker, "Speed-dependent chemotactic precision in marine bacteria," *Proceedings of the National Academy of Sciences*, vol. 113, pp. 8624–8629, Aug. 2016.

[156] G. M. Viswanathan, S. V. Buldyrev, S. Havlin, M. G. E. da Luz, E. P. Raposo, and H. E. Stanley, "Optimizing the success of random searches," *Nature*, vol. 401, pp. 911–914, Oct. 1999.

[157] H. Huo, R. He, R. Zhang, and J. Yuan, "Swimming *Escherichia coli* explore the environment by Lévy walk," *Appl. Environ. Microbiol.*, vol. 87, pp. e02429–20, Jan. 2021.

[158] M. Theves, J. Taktikos, V. Zaburdaev, H. Stark, and C. Beta, "A Bacterial Swimmer with Two Alternating Speeds of Propagation," *Biophysical Journal*, vol. 105, pp. 1915–1924, Oct. 2013.

[159] J. D. Antani, A. X. Sumali, T. P. Lele, and P. P. Lele, “Asymmetric random walks reveal that the chemotaxis network modulates flagellar rotational bias in Helicobacter pylori,” *eLife*, vol. 10, p. e63936, Jan. 2021.

[160] N. W. Frankel, W. Pontius, Y. S. Dufour, J. Long, L. Hernandez-Nunez, and T. Emonet, “Adaptability of non-genetic diversity in bacterial chemotaxis,” *eLife*, vol. 3, Oct. 2014.

[161] D. R. Brumley, F. Carrara, A. M. Hein, Y. Yawata, S. A. Levin, and R. Stocker, “Bacteria push the limits of chemotactic precision to navigate dynamic chemical gradients,” *Proc Natl Acad Sci USA*, vol. 116, pp. 10792–10797, May 2019.

[162] Y. Yawata, F. Carrara, F. Menolascina, and R. Stocker, “Constrained optimal foraging by marine bacterioplankton on particulate organic matter,” *Proceedings of the National Academy of Sciences*, vol. 117, pp. 25571–25579, Oct. 2020.

[163] M. Grognot, A. Mittal, M. Mah’moud, and K. M. Taute, “Vibrio cholerae Motility in Aquatic and Mucus-Mimicking Environments,” *Appl. Environ. Microbiol.*, vol. 87, pp. e01293–21, Sept. 2021.

[164] R. Sathyamoorthy, Y. Kushmaro, O. Rotem, O. Matan, D. E. Kadouri, A. Huppert, and E. Jurkevitch, “To hunt or to rest: prey depletion induces a novel starvation survival strategy in bacterial predators,” *ISME J*, vol. 15, pp. 109–123, Sept. 2020.

[165] S. Subramanian and D. B. Kearns, “Functional Regulators of Bacterial Flagella,” *Annu. Rev. Microbiol.*, vol. 73, pp. 225–246, Sept. 2019.

[166] K. Taute, S. Gude, S. Tans, and T. Shimizu, “High-throughput 3D tracking of bacteria on a standard phase contrast microscope,” *Nature Communications*, vol. 6, Dec. 2015.

[167] P. D. Karp, R. Billington, R. Caspi, C. A. Fulcher, M. Latendresse, A. Kothari, I. M. Keseler, M. Krummenacker, P. E. Midford, Q. Ong, W. K. Ong, S. M. Paley, and P. Subhraveti, “The BioCyc collection of microbial genomes and metabolic pathways,” *Briefings in Bioinformatics*, vol. 20, pp. 1085–1093, July 2019.
