# Supplementary material for: Risk–reward trade-off during carbon starvation generates dichotomy in motility endurance among marine bacteria
Source: Nat Microbiol. 2025 May 26;10(6):1393–403. doi: 10.1038/s41564-025-01997-7 (PMC12137127; doi:10.1038/s41564-025-01997-7)
Supplement: Supplementary file 2 — Reporting Summary [file 41564_2025_1997_MOESM2_ESM.pdf]

Corresponding author(s): Roman Stocker; Johannes Keegstra

Last updated by author(s): March 19, 2025

Reporting Summary

Nature Portfolio wishes to improve the reproducibility of the work that we publish. This form provides structure for consistency and transparency in reporting. For further information on Nature Portfolio policies, see our [Editorial Policies] and the [Editorial Policy Checklist].

Statistics

For all statistical analyses, confirm that the following items are present in the figure legend, table legend, main text, or Methods section.

n/a	Confirmed	
☐	☒	The exact sample size (*n*) for each experimental group/condition, given as a discrete number and unit of measurement
☐	☒	A statement on whether measurements were taken from distinct samples or whether the same sample was measured repeatedly
☐	☒	The statistical test(s) used AND whether they are one- or two-sided<br>*Only common tests should be described solely by name; describe more complex techniques in the Methods section.*
☐	☒	A description of all covariates tested
☐	☒	A description of any assumptions or corrections, such as tests of normality and adjustment for multiple comparisons
☐	☒	A full description of the statistical parameters including central tendency (e.g. means) or other basic estimates (e.g. regression coefficient) AND variation (e.g. standard deviation) or associated estimates of uncertainty (e.g. confidence intervals)
☐	☒	For null hypothesis testing, the test statistic (e.g. *F*, *t*, *r*) with confidence intervals, effect sizes, degrees of freedom and *P* value noted<br>*Give P values as exact values whenever suitable.*
☐	☒	For Bayesian analysis, information on the choice of priors and Markov chain Monte Carlo settings
☒	☐	For hierarchical and complex designs, identification of the appropriate level for tests and full reporting of outcomes
☐	☒	Estimates of effect sizes (e.g. Cohen's *d*, Pearson's *r*), indicating how they were calculated

Our web collection on statistics for biologists contains articles on many of the points above.

Software and code

Policy information about availability of computer code

Data collection	Nikon Elements v5.02, CytExpert v2.4, Koala Acquisition & Analysis 8.5
Data analysis	Data analysis was performed in Python 3.7 or higher. Tracking was performed using Trackpy (v0.4.2 and v0.5.0). Electron microscopy image analysis was performed using imageJ v2.1.0/1.53c. FlowCytometry analysis was performed using CytExpert v2.4. DHM image reconstruction was performed using Koala Acquisition & Analysis 8.5 (Lyncee Tech). Data visualization was performed using IQTree 2.0, Matplotlib 3.5.0 and Seaborn 0.11.2. Genomic model training and testing were performed in R 4.4.2, using packages FeatureTerminatoR, 1.0.0 naivebayes 1.0.0 and caret 7.0-1. Phylogenetic tree was constructed using Phylophlan 3.0 and post-hoc phylogenetic bias testing was performed using the R package phylolm v2.6.2. All custom code is available in the Figshare repository: https://doi.org/10.6084/m9.figshare.26195339

For manuscripts utilizing custom algorithms or software that are central to the research but not yet described in published literature, software must be made available to editors and reviewers. We strongly encourage code deposition in a community repository (e.g. GitHub). See the Nature Portfolio guidelines for submitting code & software for further information.

Data

Policy information about availability of data

All manuscripts must include a data availability statement. This statement should provide the following information, where applicable:

- Accession codes, unique identifiers, or web links for publicly available datasets
- A description of any restrictions on data availability
- For clinical datasets or third party data, please ensure that the statement adheres to our policy

All data used to support statements in this manuscript, including all bacterial cell trajectories, are available through a Figshare repository: https://doi.org/10.6084/m9.figshare.26195339. This repository includes figure source data. Raw microscopy data (>6TB) can be obtained upon request. Genome accession numbers of the bacterial strains are listed in Table S1. Ocean Microbiome Database v1.1., used in this study for model prediction, is available through https://microbiomics.io/ocean/.

Research involving human participants, their data, or biological material

Policy information about studies with human participants or human data. See also policy information about sex, gender (identity/presentation), and sexual orientation and race, ethnicity and racism.

Reporting on sex and gender	n/a
Reporting on race, ethnicity, or other socially relevant groupings	n/a
Population characteristics	n/a
Recruitment	n/a
Ethics oversight	n/a

Note that full information on the approval of the study protocol must also be provided in the manuscript.

Field-specific reporting

Please select the one below that is the best fit for your research. If you are not sure, read the appropriate sections before making your selection.

☐ Life sciences ☐ Behavioural & social sciences ☒ Ecological, evolutionary & environmental sciences

For a reference copy of the document with all sections, see nature.com/documents/nr-reporting-summary-flat.pdf

Ecological, evolutionary & environmental sciences study design

All studies must disclose on these points even when the disclosure is negative.

Study description	This study investigated how a risk-reward trade-off in bacterial motility determines the motility endurance of marine bacteria. Different marine bacterial isolates were cultured and subsequently starved for carbon, and these bacterial strains were characterized using video microscopy and cell tracking, scanning electron microscopy, flow cytometry and chemical staining. The outcome of these experiments revealed a dichotomy in motile behavior during carbon starvation, and the experiments were used to train a genomic classifier that can predict the outcome in other strains not included in training.
Research sample	All samples are culturable marine bacterial isolates from different field deployments or mesocosm experiments. Experiments were performed on 26 strains from 18 species belonging to the Gammaproteobacteria class. Model testing was performed on 7 additional marine strains from 7 different species and 2 additional non-marine species.
Sampling strategy	Of the 107 available strains with motility and/or chemotaxis genes, we selected 36 strains to test for motility and growth, some of which were from the same species to encompass intra-species and inter-species variation, and all with both chemotaxis and motility genes. Of the 30 remaining strains (four strains did not grow in marine broth and two strains did not show motility during growth in marine broth), we randomly selected 26 strains to be used in this study. No statistical method was used to pre-determine sample size.
Data collection	Bacterial motility was measured using video microscopy and cell tracking (J.M.K. and S.T.Z.). Bacterial flagellation was measured using Scanning Electron Microscopy (S.T.Z.). Bacterial cell count and viability were measured using flow cytometry (S.T.Z)., optical density (J.M.K) and colony counting (J.M.K). Presence of storage compounds were tested using fluorescent stains and inspected using microscopy (J.M.K.). Single-cell mass measurements were performed using digital holographic microscopy (D.A.B).
Timing and spatial scale	The principal timescale used to assess motility endurance was 2 days as theoretical estimates indicate that most motile bacteria will encounter at least one nutrient hotspot within this timescale (see Discussion). From each culture with one strain, bacteria were

sampled from the medium immediately before starving the cells and at {1 (1), 2--4 (3), 5--9 (7), 19--24 (22), 28--32 (30), 43--48 (46)} hours after the washing protocol started, where the number in brackets refers to the weighted average of each time window, rounded to 1 h, that was used for averaging over multiple experiments. For experiments to determine the differential biomass loss and cell viability, one sampling per 24 +/- 1 h was performed for a period of 7 days.

Data exclusions	No videomicroscopy experiments were excluded from the analysis. Bacterial trajectories were inspected manually and individual trajectories that were the result of tracking errors were removed. For colony counting, only plates with 20-350 colonies were used.
Reproducibility	All the laboratory experiments were repeated three times (unless noted otherwise) , where for each repeat the cells were cultured and starved independently.
Randomization	The selection of the bacterial strains was random, apart from the criterion that they could be grown and showed motility in rich media (2216 Marine Broth). When a subset of bacterial strains was chosen for further investigation, one strain per species were chosen. For the physiological characterizations of starvation, strains from the same genus were compared (when possible).
Blinding	Blinding was not pertinent to our study because it did not include any animals and/or human research participants. In addition, blinding was not possible since many analyses were also carried out by the person in charge of sampling.

Did the study involve field work? ☐ Yes ☒ No

Reporting for specific materials, systems and methods

We require information from authors about some types of materials, experimental systems and methods used in many studies. Here, indicate whether each material, system or method listed is relevant to your study. If you are not sure if a list item applies to your research, read the appropriate section before selecting a response.

Materials & experimental systems

n/a	Involved in the study
☒	☐ Antibodies
☒	☐ Eukaryotic cell lines
☒	☐ Palaeontology and archaeology
☒	☐ Animals and other organisms
☒	☐ Clinical data
☒	☐ Dual use research of concern
☒	☐ Plants

Methods

n/a	Involved in the study
☒	☐ ChIP-seq
☐	☒ Flow cytometry
☒	☐ MRI-based neuroimaging

Plants

Seed stocks	n/a
Novel plant genotypes	n/a
Authentication	n/a

Flow Cytometry

Plots

Confirm that:

☒ The axis labels state the marker and fluorochrome used (e.g. CD4-FITC).

☒ The axis scales are clearly visible. Include numbers along axes only for bottom left plot of group (a 'group' is an analysis of identical markers).

☒ All plots are contour plots with outliers or pseudocolor plots.

☒ A numerical value for number of cells or percentage (with statistics) is provided.

Methodology

Sample preparation	Cell counting was performed by diluting cells by a factor of 100 and staining them with Syber Green (Sigma Aldrich). For samples where the dead fraction was determined, a second sample was stained with with Sytox Green (Thermofischer). Cells were stained at a final concentration of 5 microM for both stains and incubated in the dark for 10 min at room temperature.
Instrument	CytoFLEX S (Beckman Coulter, USA)
Software	CytExpert Version 2.4
Cell population abundance	The bacteria were the only cell population present in the samples.
Gating strategy	The gating settings were set to the size of the measured objects (Forward scatter FSC) and Fluorescent intensity (FITC-A)

☒ Tick this box to confirm that a figure exemplifying the gating strategy is provided in the Supplementary Information.
